# The C-Terminus of Histone H2B Is Involved in Chromatin Compaction Specifically at Telomeres, Independently of Its Monoubiquitylation at Lysine 123

**DOI:** 10.1371/journal.pone.0022209

**Published:** 2011-07-29

**Authors:** Chen-Yi Wang, Chia-Yin Hua, Hsiang-En Hsu, Chia-Ling Hsu, Hsin-Yi Tseng, Duncan E. Wright, Pang-Hung Hsu, Chih-Hung Jen, Chia-Yeh Lin, Meng-Ying Wu, Min-Daw Tsai, Cheng-Fu Kao

**Affiliations:** 1 Institute of Cellular and Organismic Biology, Academia Sinica, Taipei, Taiwan; 2 Institute of Biological Chemistry, Academia Sinica, Taipei, Taiwan; 3 Department of Life Science, Institute of Bioscience and Biotechnology, National Taiwan Ocean University, Keelung, Taiwan; 4 VGH Yang-Ming Genome Research Center, Taipei, Taiwan; Ludwig-Maximilians-Universität München, Germany

## Abstract

Telomeric heterochromatin assembly in budding yeast propagates through the association of Silent Information Regulator (SIR) proteins with nucleosomes, and the nucleosome array has been assumed to fold into a compacted structure. It is believed that the level of compaction and gene repression within heterochromatic regions can be modulated by histone modifications, such as acetylation of H3 lysine 56 and H4 lysine 16, and monoubiquitylation of H2B lysine 123. However, it remains unclear as to whether or not gene silencing is a direct consequence of the compaction of chromatin. Here, by investigating the role of the carboxy-terminus of histone H2B in heterochromatin formation, we identify that the disorderly compaction of chromatin induced by a mutation at H2B T122 specifically hinders telomeric heterochromatin formation. H2B T122 is positioned within the highly conserved AVTKY motif of the αC helix of H2B. Heterochromatin containing the T122E substitution in H2B remains inaccessible to ectopic *dam* methylase with dramatically increased mobility in sucrose gradients, indicating a compacted chromatin structure. Genetic studies indicate that this unique phenotype is independent of H2B K123 ubiquitylation and Sir4. In addition, using ChIP analysis, we demonstrate that telomere structure in the mutant is further disrupted by a defect in Sir2/Sir3 binding and the resulting invasion of euchromatic histone marks. Thus, we have revealed that the compaction of chromatin *per se* is not sufficient for heterochromatin formation. Instead, these results suggest that an appropriately arrayed chromatin mediated by H2B C-terminus is required for SIR binding and the subsequent formation of telomeric chromatin in yeast, thereby identifying an intrinsic property of the nucleosome that is required for the establishment of telomeric heterochromatin. This requirement is also likely to exist in higher eukaryotes, as the AVTKY motif of H2B is evolutionarily conserved.

## Introduction

Silent chromatin (heterochromatin) is often associated with repetitive DNA sequences near centromeres or telomeres, and plays important roles in transcriptional regulation and chromosome segregation [1], [2]. Heterochromatin has been assumed to fold into a compacted structure [3], [4], and the level of compaction can be modulated by histone modifications [5], [6]. The popular perception is that a compacted chromatin structure inhibits gene expression. However, recent studies using cryo-EM [7], [8], ESI (electron spectroscopic imaging) [9], [10], and 3C (chromosome conformation capture) [11], [12] suggest that the basic structure of active and silent chromatin during interphase is formed by extended 11 nm nucleosome arrays instead of compacted 30 nm fibers, as was previously suggested [7], [8], [13]. Intriguingly, the incubation of purified SIR proteins with purified yeast chromatin is shown to promote the *in vitro* formation of a heterochromatin structure based on extended 11 nm fibers [14]. These observations imply that the formation of heterochromatin could occur without chromatin compaction. The precise structure of heterochromatin and the mechanism of gene silencing continue to remain elusive.

Studies in yeast, fly and mammals have suggested divergent mechanisms for the assembly of heterochromatin, but there are certain analogous features in the repressive mechanisms in these organisms [1], [2], [15]. One common theme is that heterochromatin mediated gene silencing can spread along chromosomes [5]. For example, HP1 is implicated in driving heterochromatin assembly in fly and mammals. HP1 is shown to bind to nucleosomes methylated at histone H3 K9. HP1 in turn recruits a histone methyltransferase, Suv39, that specifically methylates H3 K9 of adjacent nucleosomes. This promotes further HP1 binding, thereby leading to an iterative cycle that enables the spreading of heterochromatin [16], [17], [18], [19]. Telomeric heterochromatin in budding yeast propagates from a nucleation process via Rap1 binding at chromosome tips. Rap1 in turn recruits the silent information regulator (SIR) complex [20]. The Sir2 subunit then deacetylates histones H3 and H4 of neighboring nucleosomes, promoting additional SIR complex binding [21], [22], [23]. This initiates recurrent rounds of histone deacetylation and SIR binding, leading to the spreading of silenced chromatin.

The SIR complex is able to associate with specific nucleosomes within silent chromatin, but the molecular mechanism of how this association occurs is poorly understood. The binding sites of SIR are proposed to be formed by the highly conserved N-terminal tails and globular domains of H3 and H4 [5], [20], [24], [25]. Deacetylation of H4 K16 in the H4-N terminus is particularly crucial for Sir3 binding *in vivo* and *in vitro* [26], [27], [28]. Besides acetylation, histone methylation is involved in regulating the spreading of silent chromatin in budding yeast. H3 K4 and K79 methylations catalyzed by Set1 or Dot1 respectively are thought to prevent promiscuous binding of SIR at loci other than the sub-telomeric regions [29].

In addition to H3 and H4 N-termini, the conserved H2B C-terminus also contributes to telomeric silencing [30]. The crystal structure of the yeast nucleosome core particle predicts that inter-nucleosomal contacts are made by the H2B αC helix (hereafter simplified as H2B αC) because this extremely well ordered H2B αC is crucial in defining the surface of the nucleosome [31]. The sole modification identified at H2B αC is the monoubiquitylation of lysine 123, located at the highly conserved AVTKY motif [32]. As such, the dynamic regulation of H2B K123 ubiquitylation (H2Bub1) serves as a good candidate to shape chromatin structure, by modulating inter-nucleosomal interactions [33]. However, it is not known whether the H2B αC has a *bona fide* function in regulating SIR binding and higher-order organization of silent chromatin.

Here, we have investigated the role of the H2B αC in the assembly of heterochromatin *in vivo*, through the use of yeast strains that carry mutations in the residues of H2B αC. Our experiments using genetic analysis, bacterial *dam* methylase access and sucrose gradient sedimentation, all indicate a unique role of H2B αC in silent chromatin assembly, independent of H2Bub1. Surprisingly, we find that telomeric chromatin is assembled into a nucleosomal array with a regular alignment that requires H2B T122. The replacement of H2B T122 with glutamic acid induces disorderly chromatin compaction specifically at the telomere, and invasion of euchromatic histone marks. The results suggest that the organization of telomeric chromatin may be based on an extended chromatin fiber *in vivo*.

## Results

### H2B T122 regulates telomeric silencing

**Figure 1:**
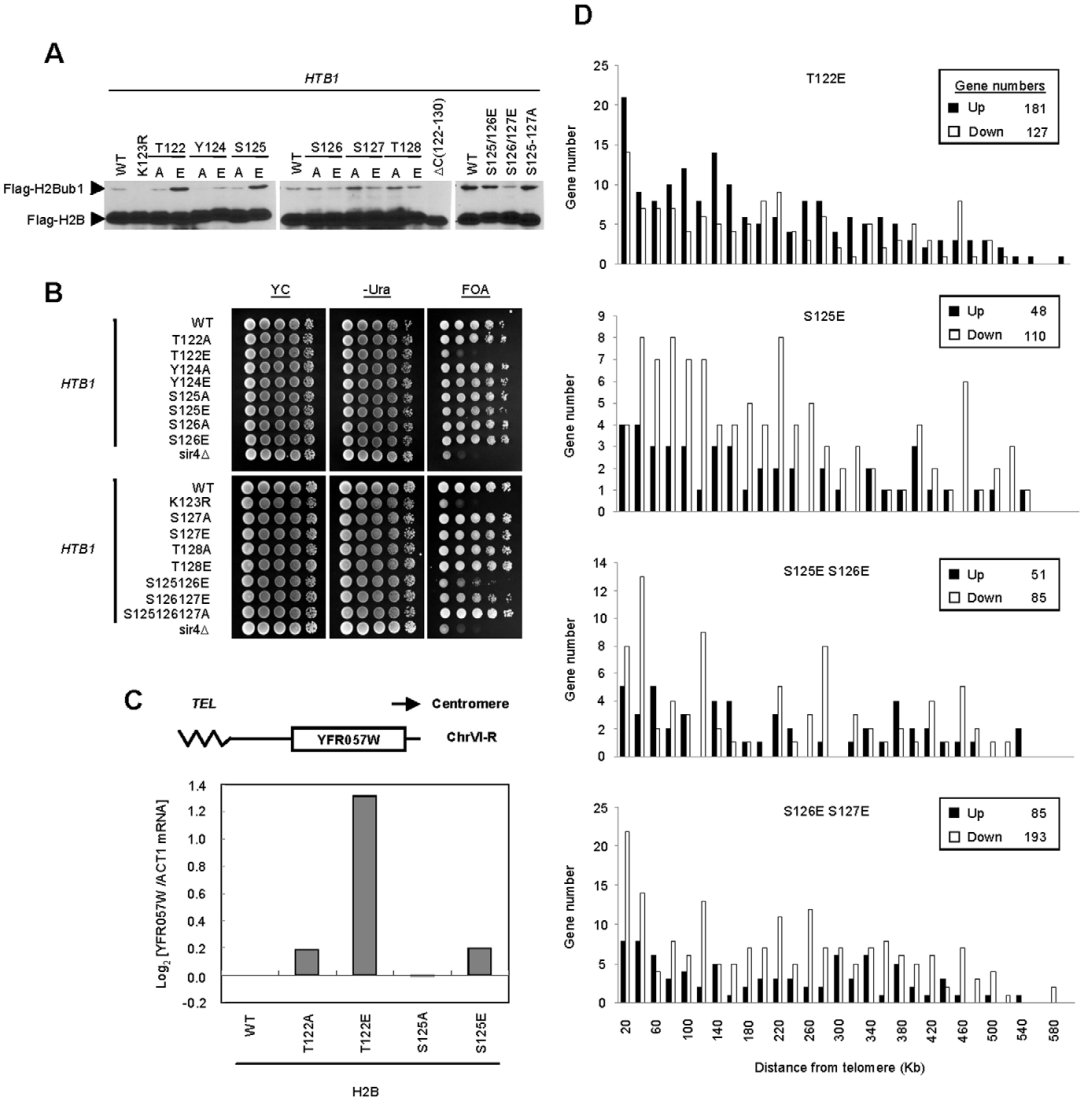
The effects of H2B C-terminus on the level of H2Bub1 and telomere silencing. (*A*) Western blot analysis of whole-cell extracts using anti-Flag antibody shows the level of H2B (Flag-H2B) and its ubiquitylated form (Flag-H2Bub1) in the indicated strains (derived from Y131) expressing Flag-*HTB1* or Flag-*htb1* mutants. (*B*) Telomere silencing analysis of the indicated strains (derived from UCC6389) expressing *HTB1* or *htb1* mutants. Cells were 10-fold serially diluted and spotted on YC with or without uracil (-Ura), or YC containing FOA (FOA). The strain *sir4Δ* was used as a control for the defect of telomere silencing. (C) The mRNA level of an endogenous gene near a telomere, *YFR057W*. Total RNA was extracted from the yeast strains, reverse transcribed and then quantified by real-time PCR using the primer pairs against *YFR057W* sequences. The obtained signals were normalized with the signal from *ACT1*, and then the value of WT was taken as 1 before log transformation. (*D*) Transcript array analysis was performed using strains (derived from Y131) expressing *htb1-T122E*, *S125E*, *S125E/S126E*, and *S126E/S127E*. Transcripts were isolated and analyzed by Phalanx Yeast OneArray®. Numbers of genes affected in the *htb1* mutants compared to WT plotted by their positions from telomeres. The numbers of up-regulated genes are represented by the black bar; down-regulated genes are represented by the white bar.

The H2B αC, which contains the ubiquitylation site (K123) within the highly conserved AVTKY motif [32], is enriched with residues that may be targeted for phosphorylation (T, Y and S). We hypothesized that the phosphorylation of the residues surrounding K123 may play a *cis*-regulatory role on its ubiquitylation level. To determine whether or not this were the case, we systematically mutated the residues T122 to T128 to alanine (A), thereby preventing phosphorylation, or glutamic acid (E), mimicking the phosphorylated state of these residues (Table S1). Consistent with a possible role for phosphorylation, we found that replacing T122 and S125 with glutamic acid elevated levels of H2B K123 ubiquitylation (Fig. 1A). The increased levels of H2Bub1 in *htb1-T122E* (*HTB1* encodes H2B) and *htb1-S125E* cells were comparable to that of a *ubp8*Δ strain [34] (Fig. S1). To determine whether or not phosphorylation occurs on T122 or S125, we isolated H2B and ubiquitylated H2B from acid extracted histones and attempted to identify post translational modifications using mass spectrometry. We reasoned that if there is cross-talk between ubiquitylation and phosphorylation, the latter modification may be detectable under conditions of elevated H2Bub1. Through the course of this study, we found that treatment of cells with HU and H_2_O_2_ elevates H2Bub1 (data not shown). After several attempts under both conditions of basal and increased H2Bub1 (Table S2), we were unable to detect phosphorylation at T122 or S125, while phosphorylation occurring at T39 and T128 of H2B was identified repeatedly. The data suggested that the increased levels of H2Bub1 observed in *htb1-T122E* and *htb1-S125E* cells are not related to the putative phosphorylation of H2B T122 or S125. The increase could be a direct consequence of the reduced accessibility of the H2B specific ubiquitin proteases, Ubp8 or Ubp10, to chromatin as recently proposed by Sun and colleagues [35].

The balanced level of H2Bub1 is essential for maintaining silencing at telomeric chromatin [36], [37]. Thus, we anticipated that both *htb1-T122E* and *htb1-S125E* mutants may display defects in telomeric silencing. To investigate this, we used yeast strains with the reporter gene *URA3* inserted at a position 1.3 Kb away from a telomere located at chromosome VII, which causes it to be silenced. The activation of *URA3* can convert 5-Fluoroorotic Acid (5-FOA) into toxic 5-fluorouracil causing cell death. We tested whether telomeric silencing was disrupted in any of the strains in the mutation library by growing them on 5-FOA containing plates. In addition to the previously reported telomeric silencing defects of *htb1-K123R* and *sir4*Δ [30], [38], [39], the *htb1-T122E* strain also exhibited strong activation of *URA3* gene as evidenced by extremely poor growth on 5-FOA plate, while surprisingly the *htb1-S125E* strain showed no defects in telomeric silencing, despite the increase in H2Bub1 observed (Fig. 1B). Double mutants carrying glutamic acid substitutions at S125/126 or S126/127 of H2B also mildly compromised cell survival on 5-FOA plates. Besides telomere, the silencing effect on mating loci was also analyzed by the pheromone halo assay [40]. In this assay, the area surrounding the filters soaked with α factor result in a zone of growth inhibition of cells in which the mating loci are silent. If the silencing effect at mating loci is disrupted, as in *SIR3* null mutants, cells are resistant to growth inhibition by α factor and the clear zone surrounding the filter will disappear (Fig. S3A). Interestingly, the clear zone was seen in most strains tested, including *htb1-T122E*, implying that this mutant affects the expression of genes near the telomere but not at *HML*.

In addition to the reporter assay and the pheromone halo assay, we measured the transcriptional activity of an endogenous gene, *YFR057W*, positioned 1.5 Kb away from a telomere at chromosome VI and *HML α1* located at *HML* locus. Derepression of *YFR057W* but not *HML α1* was observed in *htb1-T122E* (Fig. 1C and S3B), consistent with our observations in Fig. 1B and S3A. To eliminate the possibility that the observed telomere specific effect of *htb1-T122E* is due to Sir proteins relocalization from the telomeres to the mating loci (thereby enhancing silencing at the latter), we used chromatin immunoprecipitation to investigate the localization of Sir proteins at *HML* region. Several sites located at the left arm of Chr. III have been analyzed, including the E silencer, *HML α1* and its promoter region. *SPS22*, which is about 3 kb away from *HML* locus, was taken as a control. As shown in Fig. S3C, Sir2 levels in *htb1-T122E* were similar with that in wild-type and *htb1-T122A* cells at all the three sites. Overall, we conclude that the effect of the *htb1-T122E* mutation in gene silencing is specific to the telomeres.

H2Bub1 is known to be a prerequisite for methylation of K4 and K79 of H3 [30], [41], both of which contribute to telomeric silencing [42], [43]. As such, we then inspected the methylation levels of H3 K4 and H3 K79 in the mutant library strains. We did not observe significant changes in all histone modifications examined (Fig. S2). Thus, the telomeric silencing defect we observed in *htb1-T122E* cells may not be a consequence of changes in H3 methylations.

To determine if the mutations in the H2B C-terminus have a general role in telomere silencing, we performed genome-wide transcriptional profiling of H2B mutant cells (Fig. 1D). After applying vigorous statistical criteria (p<0.05 & differential expression of at least 1.5 fold), we found that the genes located near the end of chromosomes tend to be de-repressed in *htb1-T122E* cells (Fig. 1D), while the location of de-repressed genes in *htb1-S125E* and other mutant cells (*S125/125E* and *S126/127E*) showed no bias in their proximity to telomeres (Fig. 1D). Taken together, the discrepancy in the effect on telomere silencing between *htb1-T122E* and *htb1-S125E* is demonstrated by three independent assays.

### Heterochromatin containing htb1-T122E forms a condensed structure

**Figure 2:**
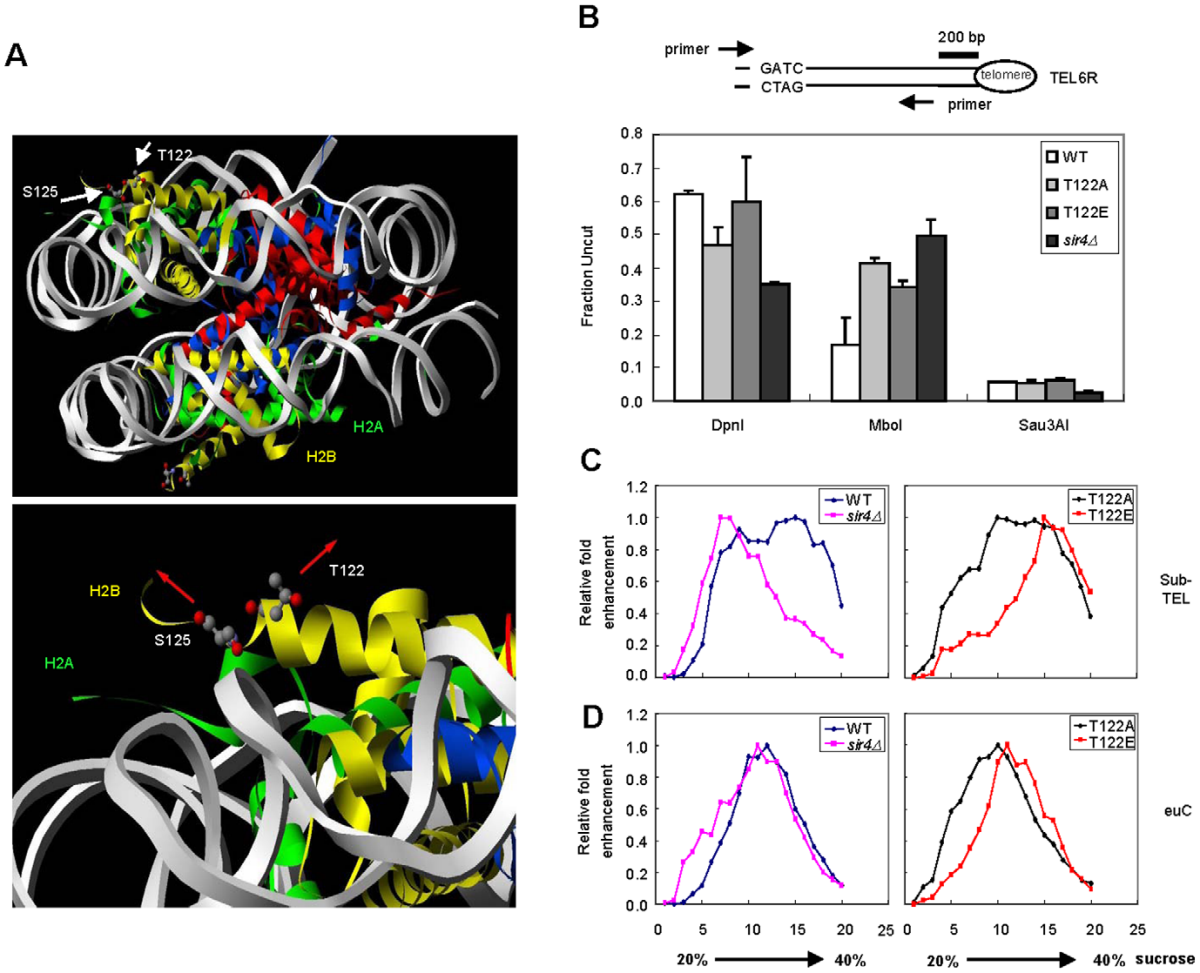
Silent chromatin in *htb1-T122E* is less accessible and more compact. (*A*) Stereo view of T122 and S125 of H2B at the yeast nucleosome core particle. The nucleosome is viewed down the dyad axis. Histones and DNA are indicated by ribbons of the following colors: H2A is shown in green, H2B in yellow, H3 in red, H4 in blue, and the DNA in grey. In the upper panel, the white arrows denote the positions of T122 and S125 of H2B with respect to the surface of the nucleosome disk. A magnified view of the H2B αC with the T122 and S125 side chains is shown in the lower panel. The red arrows indicate the directions in which the side chains point. (*B*) Analysis of DNA methylase accessibility in the indicated yeast strains derived from UCC6389 (*HTB1* WT, *htb1-T122A*, *htb1-T122E*, and *sir4Δ HTB1* WT) expressing the *E. coli dam* methylase. The diagram shown in the upper panel indicates the enzyme cutting site (GATC) and the relative locations of primers specific to the right end of Chr. VI. (*C*) Hydrodynamics of yeast chromatin fragments were determined by mobility in a 20%–40% sucrose gradient upon ultracentrifugation. The relative positions of the fragments containing the sub-telomeric (sub-TEL) or euchromatin region (euC) are shown in (*C*) or (*D*), respectively. The results for strains expressing *HTB1* (derived from UCC6389), and *sir4Δ* (strain UCC6391) are shown in the left panel; results for strains expressing either *htb1-T122A* or *htb1-T122E* are shown in the right panel.

The crystal structure of the yeast nucleosome suggests that the H2B C terminus is exposed on the surface of the nucleosome core particle [31], and as such, it may have a potential role in regulating nucleosome-nucleosome interactions. H2B T122 faces towards the surface of the nucleosome disk and the side chain of H2B S125 points in the opposite direction (Fig. 2A) [31]. H2B T122 may play an essential role in maintaining telomeric heterochromatin due to its unique position at the H2B C terminus. We proposed that the substitution of T122 with glutamic acid may cause an alteration in nucleosome compaction at the telomere. To test the hypothesis, we set out to determine whether the *htb1-T122E* mutation might cause chromatin to become more accessible to DNA binding proteins. To analyze this *in vivo*, we used a bacterial DNA methylase accessibility assay [44]. The genomic DNA from wild type and mutant cells containing an integrated, constitutively expressed *dam* methylase gene were extracted and subjected to restriction enzyme digestion. Three enzymes were used to digest methylated (DpnI), unmethylated (MboI) or both methylated and unmethylated (Sau3AI) GATC sites (Fig. 2B). We then measured the degree of digestion at the end of telomere VIR using primers that cross the digested sites in quantitative real-time PCR. We found that loss of Sir4 leads to an increase of *dam* methylase accessibility at a subtelomeric region as compared to wild type (Fig. 2B), consistent with previous reports [44]. Surprisingly, the DNA extracted from *htb1-T22E* cells was digested by DpnI to a similar extent as the wild type DNA, suggesting that chromatin containing *htb1-T122E* is as inaccessible to *dam* methylase as that in wild type cells (Fig. 2B). This implies that the telomeric chromatin structure in *htb1-T122E* cell is distinctive from that of a *SIR4* deletion strain, although both conditions lead to disruption of telomeric silencing.

To elucidate whether the telomeres in *htb1-T122E* cells carry a special form of chromatin, we made use of sucrose gradient sedimentation to assay the hydrodynamic properties of silent chromatin in WT and mutant cells, as demonstrated recently [24]. We selected two ∼10 Kb chromatin segments located at right sub-telomeric (sub-TEL) and euchromatic (euC) regions (map in Fig. S4) respectively within chromosome III for analysis. These two regions share a common property in that they are both blocks of ∼10 Kb continuous chromatins flanked by BglII restriction enzyme sites. These two chromatin segments carry a similar mass, so their mobility in the sucrose gradient is determined by their conformation and density. Yeast nuclei extracted from WT and mutant strains were prepared and digested with BglII enzyme. The efficiency of the digestion (65–70%) was confirmed by quantitative PCR using two primer pairs flanking each of the two BglII sites located in *HML* as shown previously [24] (data not shown). The soluble fraction of BglII digested chromatin was separated by a 20–40% sucrose gradient. The relative positions of the two chromatin segments within each fraction were measured by quantitative PCR using primers specific to each of the chromatin segments (Fig. S4). Previous studies have shown that the heterochromatin from mutant cells with a decondensed structure exhibits a migration profile shifted towards the 20% fraction of the sucrose gradient [24]. As expected, sub-TEL from *sir4*Δ cells migrated less than the same fragments isolated from wild type cells (Fig. 2C), whereas euC of *sir4*Δ migrated to a similar density as WT (Fig. 2D), indicating that Sir4 is specifically involved in organizing chromatin structure in telomere. As telomeric chromatin bearing *htb1-T122E* is unable to repress gene activity, we may predict a more open telomeric heterochromatin structure within these cells as well, and as such we expected that the segments of telomeric chromatin from *htb1-T122*E cells would distribute towards the 20% end of the sucrose gradient. On the contrary, sub-TEL chromatin from *htb1-T122E* (but not *htb1-T122A*) cells migrated through the sucrose gradient towards the 40% side, while euC from all cells exhibited similar migration patterns (Fig. 2D). This striking result argues that the *htb1-T122E* substitution transforms silenced chromatin into a more condensed structure, and yet counter intuitively specifically impairs telomeric silencing. To our knowledge, this is the first demonstration that chromatin compaction can be uncoupled from telomeric silencing.

### H2B T122 maintains chromatin structure at the telomere independently of H2B ubiquitylation

**Figure 3:**
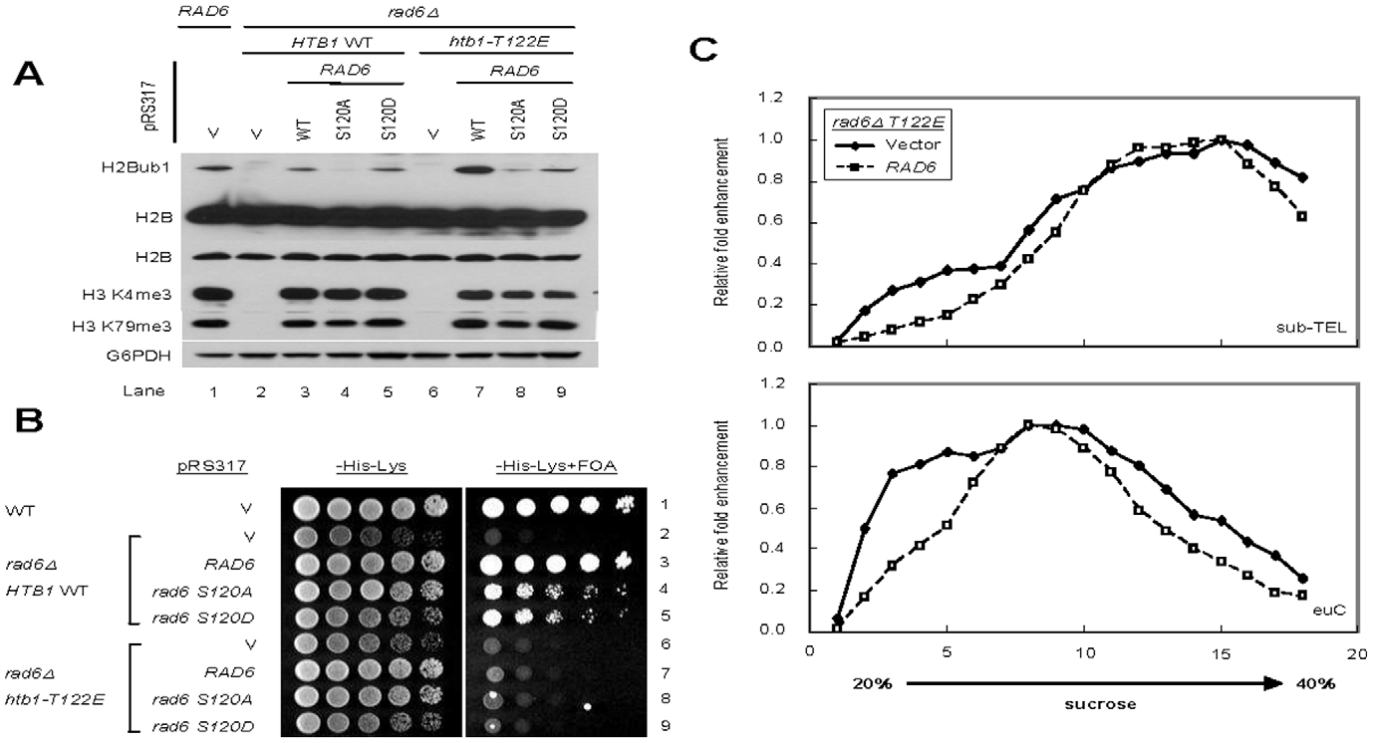
Aberrant chromatin compaction induced by *htb1-T122E* is independent of H2Bub1. (*A*) Plasmids carrying *RAD6* or *rad6* mutants were transformed into the strains UCC6389 with *RAD6* deletion expressing *HTB1* or *htb1-T122E*. WCEs prepared from the indicated strains were analyzed by western blot. H2B and its ubiquitylation were detected by anti-Flag antibody; H3 K4 and K79 trimethylation were analyzed using anti-H3 K4me3 or anti-H3 K79me3 antibodies. The G6PDH antibody was used to monitor protein loading. (*B*) Overnight cultures of the indicated strains were 5-fold serial diluted and spotted on the indicated plates (-His-Lys for plasmid selection; -His-Lys+FOA for telomere silencing analysis). (*C*) H2Bub1 does not affect chromatin condensation in *htb1-T122E* cells, as shown by sucrose gradient centrifugation. Vector only or plasmids with WT *RAD6* were transformed into *rad6Δ htb1-T122E* yeast with UCC6389 background. The relative positions of the fragments containing sub-telomeric (sub-TEL), or euchromatin region (euC), were shown in upper panel or lower panel, respectively.

Since both *htb1-T122E* and *htb1-S125E* mutant cells exhibited increased levels of H2Bub1 but only the former substitution affects silencing at telomeric heterochromatin, it is possible that *htb1-T122E* may exert its effect on telomere chromatin independently of H2B ubiquitylation. Were this to be the case, a decrease in the level of H2Bub1 would not be able to restore silencing in the presence of the *htb1-T122E* mutation. To test this, we used a genetic manipulation to bring down the level of H2Bub1 in *htb1-T122E* and *htb1-S125E* cells. The E2 enzyme for H2Bub1, Rad6, is phosphorylated at S120 [45], [46]; when it is replaced by alanine or aspartic acid, the level of H2Bub1 in the cells is reduced (Fig. 3A). In the *rad6-S120A/D* mutant, telomeric silencing is only marginally affected (Fig. 3B). Taking advantage of the *rad6-S120A/D* mutation, we found that double mutant strains carrying a combination of *rad6-120A/120D* with *htb1-T122E* (lane 8 and 9, Fig. 3A) or *htb1-S125E* (lane 8 & 9, Fig. S5A) conferred a level of H2Bub1 equivalent to or less than that in WT cells (lane3, Fig. 3A) with no significant change in the levels of methylated H3 K4 and K79. Interestingly, telomeric silencing in *htb1-T122E/rad6-S120A or htb1-T122E/rad6-S120D* cells was still dramatically disrupted (row 8 and 9, Fig. 3B). On the other hand, the *RAD6* mutants only exhibited minor additional defects in telomeric silencing when combined with *htb1-S125E* (row 8 and 9, Fig. S5B).

Next we asked whether the increased mobility of the telomeric chromatin of *htb1-T122E* in sucrose gradient is due to the increased level of H2Bub1 in the cell. To address this question, we have used the strains *htb1-T122E*/*rad6Δ* (with no H2Bub1; lane 6, Fig. 3A) and *htb1-T122E*/*rad6Δ* supplemented with *RAD6* (the latter restoring levels of H2Bub1; lane 7, Fig. 3A) to perform the same analysis (Fig. 3C). The major difference in chromatin mass between *htb1-T122E*/*rad6Δ* with or without *RAD6* is the ubiquitin moiety on H2B. As shown in Fig. 3C, we found that there was no difference between the mobility of either sub-telomeric chromatin or euchromatin fragments; the distribution of the telomeric heterochromatin in sucrose gradient sedimentation is similar in both *htb1-T122E*/*rad6Δ* with or without *RAD6*, implicating that the increased mass of chromatin introduced by the additional ubiquitin molecules do not play a major role in increasing the mobility of telomeric heterochromatin of *htb1-T122E*. Crucially, the results also suggest that the mobility of the chromatin of *htb1-T122E* in sucrose gradient sedimentation is mainly due to the effect of *htb1-T122E* on chromatin conformation (Fig. 2C and 3C). Taken together, these results suggest that the elevated H2Bub1 is not the direct cause of the defect in telomeric silencing and the chromatin compaction in *htb1-T122E* cells. The highly conserved H2B T122 residue may have a role in maintaining heterochromatin structure independently of its effect on H2B ubiquitylation.

### H2B T122 is required for telomeric chromatin formation

**Figure 4:**
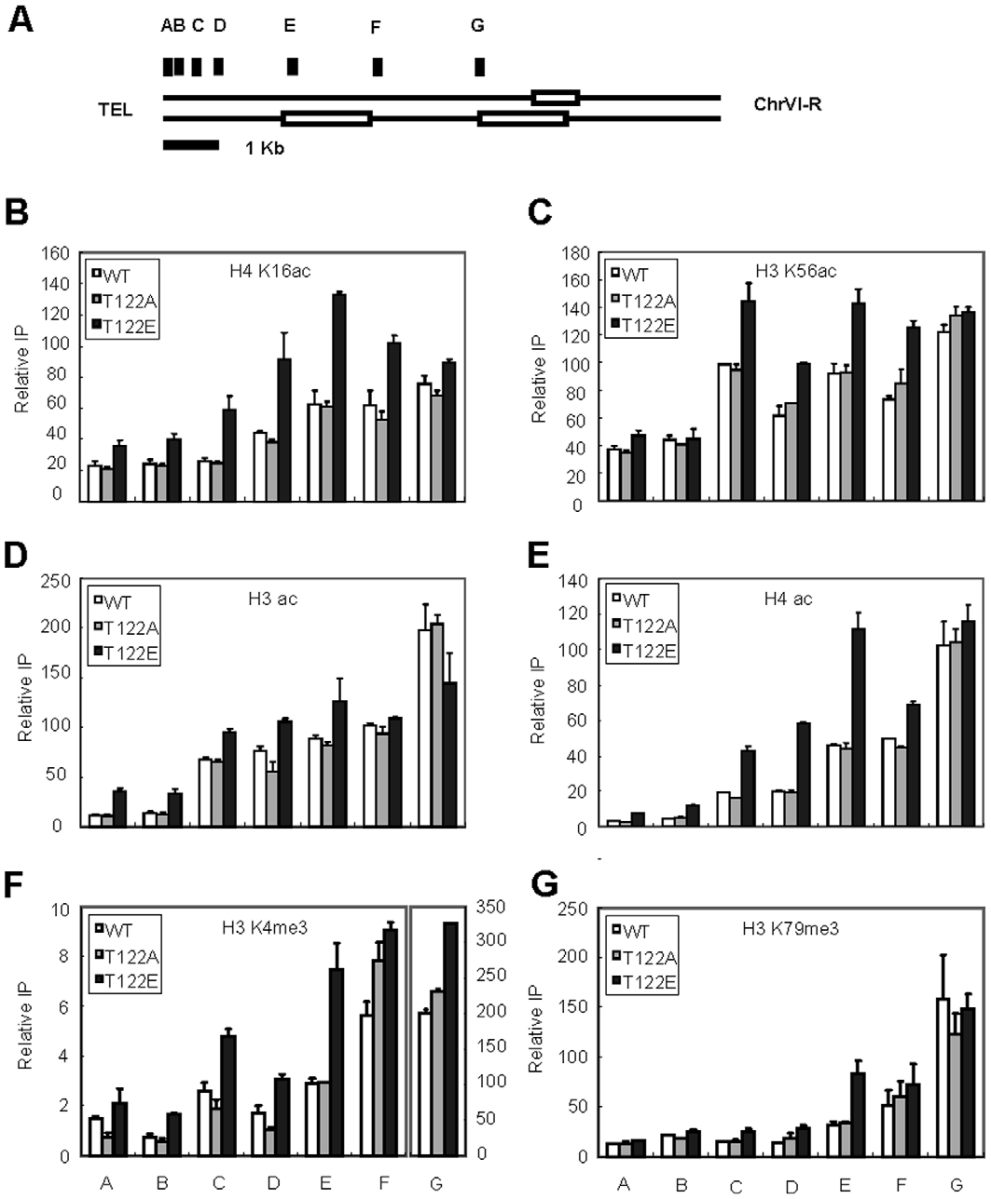
Chromatin harboring *htb1-T122E* has characteristics of euchromatin. Strains derived from Y131 expressing *HTB1*, *htb1-T122A* and *T122E* were analyzed by chromatin immunoprecipitation and then detected by quantitative PCR. Primer pairs against the indicated regions are shown in (*A*). (*B*–*G*) The distribution of histone modifications on the subtelomeric region was determined by immunoprecipitation using the indicated antibodies (*B*) α-H4 K16ac (*C*) H3 K56ac, (*D*) H3 ac, (*E*) H4 ac, (*F*) H3 K4me3, and (*G*) H3 K79me3 antibodies. At least two independent ChIP experiments were done and representative figures are shown. Values are averages of three independent real-time PCR with error bars shown for standard deviations.

The action of histone deacetylation by Sir2 is crucial for telomeric silencing [28], [47]. Of particular importance, H4 K16 acetylation is shown to inhibit chromatin compaction and gene silencing [48]. To investigate the properties of telomeric chromatin harboring *htb1-T122E*, we determined the histone acetylation status of the two Sir2 target sites: H4 K16 [26], [27] and H3 K56 [49] by ChIP, at the loci shown in Fig. 4A. Unexpectedly, despite the more condensed structure in *htb1-T122E* cells, H4 K16 acetylation was increased about 2-fold at telomeric regions and H3 K56 acetylation was also increased (Fig. 4B and 4C). Acetylation at these two sites is associated with actively transcribed euchromatin, and so we predicted that additional marks of euchromatin may be present in the telomere of *htb1-T122E* cells. Upon ChIP of such active chromatin marks, it became evident that H3 and H4 acetylation [50], [51], invaded into the proximal telomeric regions (Fig. 4D and 4E). In addition, we observed a 2-fold increase in H3 K4 methylation along with a mild increase in H3 K79 methylation at telomeric chromatin in *htb1-T122E* cells (Fig. 4F and 4G). These results suggest that even with a more condensed structure, telomeric heterochromatin containing *htb1-T122E* becomes more accessible to histone modifying enzymes that actively mark euchromatin.

**Figure 5:**
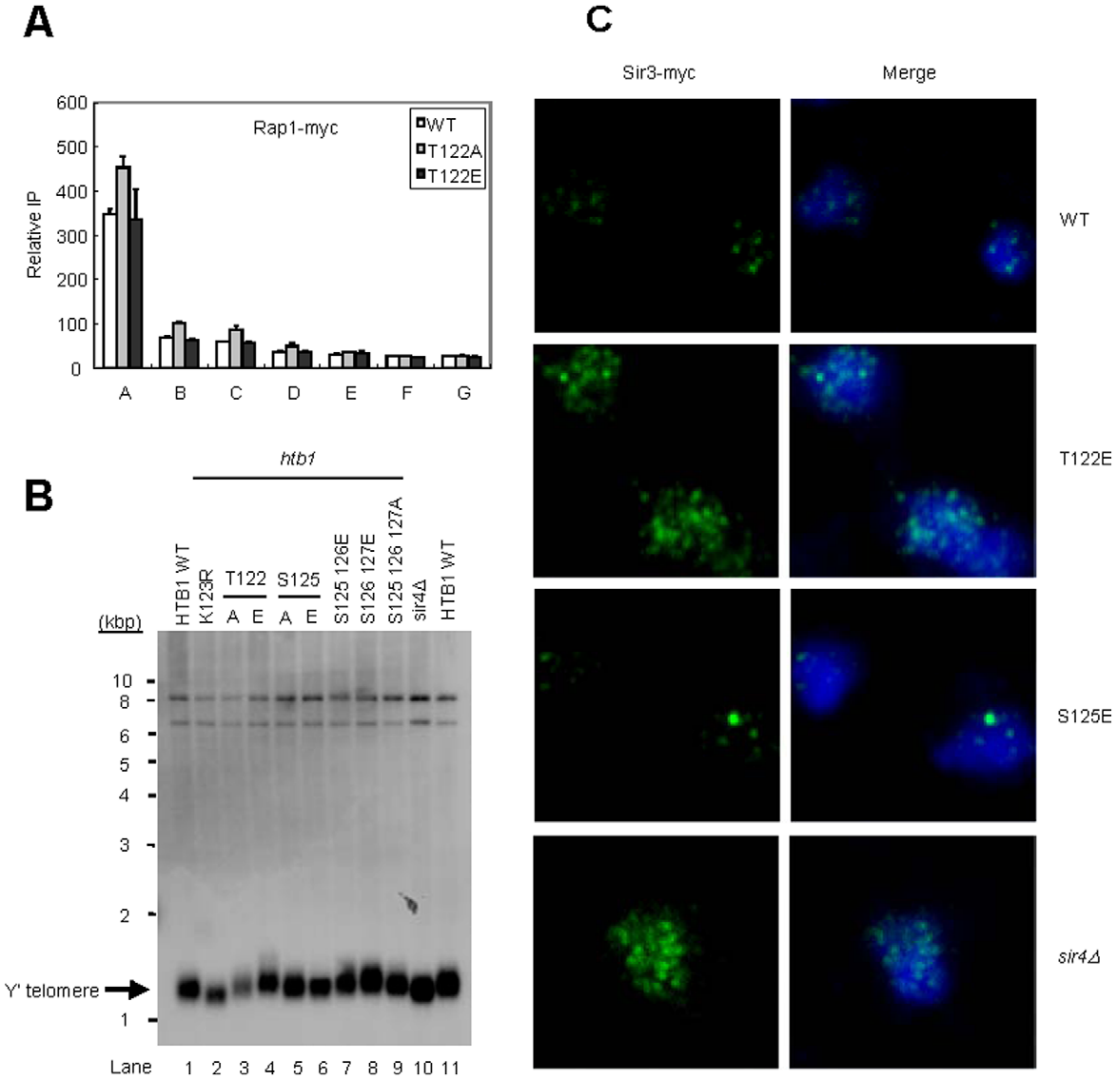
*htb1-T122E* disrupts telomere clustering. (*A*) Rap1 distribution on the end of Chr. VI-R in the strains derived from Y131 with Rap1-myc expressing *HTB1* WT, *htb1-T122A* and *htb1-T122E* was analyzed by chromatin immunoprecipitation and then detected by quantitative PCR. (*B*) Telomere length determination in the indicated strains derived from UCC6389. Genomic DNA were extracted, digested with *Xho*I, and analyzed by Southern blot using probes specific to Y′-telomere DNAs. (*C*) Indirect immunofluorescence of telomere ends in yeast strains derived from Y131 expressing *HTB1* WT, *htb1-T122E*, or *htb-S125E*, or with *sir4Δ*. All strains carry a myc-tagged *SIR3* allele in the genome for monitoring telomere foci. Cells fixed and permeabilized on glass slides were decorated with mouse α-myc monoclonal antibodies (for Sir3) and antibody complexes were later bound with Alexa Fluor 488 goat α-mouse IgG antibodies for visualization. Nuclei were stained with DAPI. Sir3 is in the green channel (left); DAPI is in the blue channel (right).

Telomeric heterochromatin in budding yeast propagates from a nucleation process via Rap1 binding at chromosome tips, and involves the association and spreading of Silent Information Regulator (SIR) proteins onto a nucleosome array. To define the molecular mechanism behind the telomeric silencing defect of *htb1-T122E* cells, we first examined the nucleation step of silent chromatin formation [20] by measuring the binding of Rap1 and the length of the repetitive sequences at a representative telomere (Chr. VI-R). We found that Rap1 appears to bind normally (Fig. 5A). In addition, we found that telomere length in *htb1-T122E* cells was similar with that in wild type, whereas *htb1-K123R* and *sir4*Δ cells had shorter telomeres (Fig. 5B). These results indicate that H2B T122 plays no major role in nucleating chromatin assembly at telomere.

Following nucleation, telomere silencing in budding yeast is achieved by the clustering of telomeres near the nuclear periphery [52], [53] where Sir3 of the SIR complex binds to both N-terminal histone tails and the LRS (loss of rDNA silencing) domain on the surface of the nucleosome [54], allowing the SIR complex and silencing to spread. To test whether *htb1-T122E* disrupts telomeric silencing by interfering with the establishment of telomere clustering, we next performed immunofluorescence experiments using anti-myc antibody to recognize C-terminal myc-tagged Sir3 and analyzed the clustering patterns by confocal laser microscopy (Fig. 5C and Fig. S6). In wild type cells, Sir3 formed punctuated foci within the nucleus, in which bright spots were typically observed above background and that often appeared in regions surrounding the nuclear periphery, presumed to pertain to telomere clusters [52]. On the contrary, Sir3 distribution in a *sir4* null mutant was dispersed over the entire nuclei. In *htb1-T122E* mutants, diffused nuclear Sir3-myc staining was observed, as seen in *sir4*Δ mutant cells (Fig. 5C and Fig. S6), while Sir3 distribution in *htb1-S125E* appeared to be similar to wild type. Thus, Sir3 is dissociated from the telomere clusters in *htb1-T122E* cells but not in *htb1-S125E* cells, consistent with the defect of telomere silencing observed previously (Fig. 1B and 1C). Although the basis for this dissociation is not clear at this stage, it is likely that the telomeres in *htb1-T122E* cells are still associated with the nuclear envelope, because the length of telomere is not shortened and Rap1 still binds normally to the end of the chromosome, both of which are required for telomere anchoring [53]. Taken together, we predict that the dispersed Sir3 in *htb-T122E* cells may be due to compromised SIR binding to telomere.

### H2B T122 play a dominant role in telomeric chromatin formation

**Figure 6:**
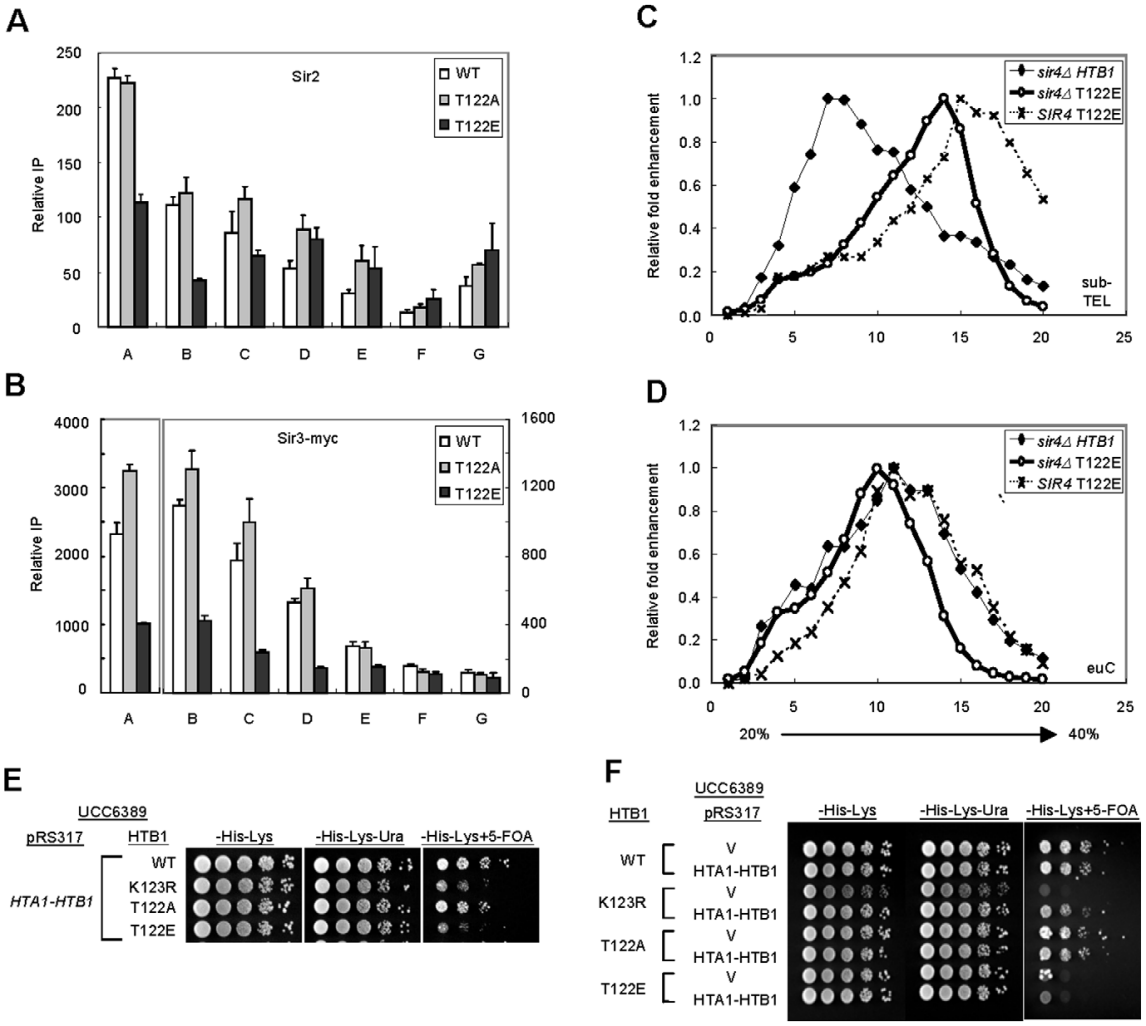
*htb1-T122E* dominantly impairs telomeric chromatin formation. (A–B) The effects of H2B C-terminal mutations on association of SIR proteins in telomeric regions. Strains derived from Y131 expressing *HTB1*, *htb1-T122A* and *T122E* were analyzed by chromatin immunoprecipitation using α-Sir2 Ab (*A*) or α-myc Ab to detect myc-tagged Sir3 (*B*). (*C–D*) The effect of T122E on chromatin mobility is SIR complex independent. Distribution of chromatin from a strain derived from UCC6389 expressing *htb1-T122E* and strains derived from UCC6391 (*sir4Δ*) expressing *HTB1* (or *htb1-T122E*) were analyzed by 20%–40% sucrose gradient ultracentrifugation. The relative positions of the fragments containing sub-telomeric (sub-TEL), or euchromatin region (euC), were shown in (*C*) or (*D*), respectively. (*E–F*) The dominance of *htb1* mutants was analyzed by telomere silencing assay. Plasmids carrying *HTB1* or *htb1* mutants were transformed into strain UCC6389 expressing *HTB1* (*E*), or plasmids carrying *HTB1* were transformed into the strain UCC6389 expressing *HTB1* or *htb1* mutants (*F*). Overnight cultures of the indicated cells were 5-fold serial diluted on the indicated plates.

We next investigated if H2B T122 is required for the recruitment of SIR at the telomere. Indeed, we observed that *htb1-T122E* caused a 2–3 fold reduction in Sir2 and Sir3 levels at the telomere-proximal regions as compared to WT and *htb1-T122A* cells (Fig. 6A and 6B), whereas Sir3 binding at the telomere in *htb1-S125E* cells was as that for wild type cells (Fig. S7). Global protein levels of Sir2, Sir3 and Sir4 in *htb1-T122E* cells were unchanged compared to wild type, suggesting a defect in their recruitment to telomere (Fig. S8).

We hypothesized that maybe the remaining ∼30% (relative to wild type) of Sir proteins still bound to the telomere in *htb1-T122E* (Fig. 6A & B) may associate with the nucleosome surface in an irregular manner. This in turn may cause chromatin to aggregate and behave hydrodynamically as a compact rigid rod. To test this, we performed sucrose gradient centrifugation using a double mutant, *htb1-T122E*/*sir4Δ*, and compared to cells with single mutations of *htb1-T122E* or *sir4Δ*. We found that the effect of *htb-T122E* is independent of Sir4 binding, as the mobility of double mutant *htb1-T122E*/*sir4Δ* telomere heterochromatin (sub-TEL) in the sucrose gradient was comparable to that of *htb1-T122E*, apart from a reduced signal at higher percentage fractions (Fig. 6C & D). Intriguingly, the level of H2Bub1 in the *htb1-T122E*/*sir4Δ* strain was reduced (Fig. S9) compared to that in *htb1-T122E*. This was not due to a change in the expression level of factors related to H2B ubiquitylation pathways, including *BRE1, RAD6, PAF1, UBP8 and UBP10* (Fig. S10). Thus, it might be a consequence of increased accessibility of ubiquitin proteases to chromatin upon combination of *sir4Δ* and *htb1-T122E*. As such, sucrose gradient sedimentation using chromatin from *htb1-T122E*/*sir4Δ* demonstrated the relatively minor contributions of SIR and H2Bub1 to the hydrodynamic properties of *htb1-T122E* heterochromatin. In summary, we conclude that the *htb1-T122E* mutation exhibits powerful dominant negative properties with respect to telomeric chromatin formation.

To further investigate the dominant-negative properties of *htb1-T122E* in telomeric silencing, we introduced low-copy plasmids bearing *htb1-T122A*, *htb1-T122E* or *htb1-K123R* into wild type cells. The result showed that the plasmids bearing *htb1-T122E* and *htb1-K123R* were able to assemble into chromatin and disrupt telomeric silencing, even in the presence of wild type H2B (Fig. 6E). Reciprocally, we transformed a plasmid harboring wild type *HTB1* into yeast strains containing *htb1-T122A*, *htb1-T122E* and *htb1-K123R* to test if the wild type H2B is able to compete with the mutant form of H2B and rescue the telomeric silencing defects. As shown in Fig. 6F, wild type H2B could restore telomere silencing in *htb1-K123R* cells but not in *htb1-T122E* cells. This demonstrates that *htb1-T122E* possesses dominant negative properties.

These results argue that the *htb1-T122E* substitution transforms silenced chromatin into a more condensed structure; however, it also appears to be accessible to histone modifying enzymes, on the basis of its enrichment with active histone marks (Fig. 4). This suggests that the T122E substitution in histone H2B induces an aberrant and unique chromatin structure at the telomere, and that its effect is dominant over that of Sir4 binding and H2Bub1.

## Discussion

**Figure 7:**
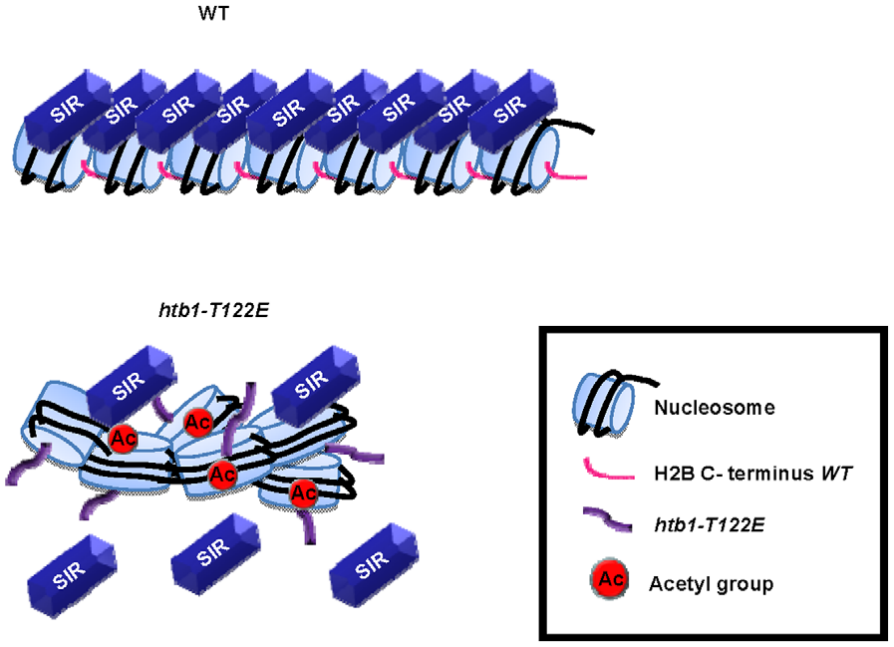
A proposed model for telomeric heterochromatin assembly. For telomeric heterochromatin assembly, it is necessary for nucleosomes to form an appropriately arrayed chromatin so that SIR complex can bind to the nucleosome stably and then deacetylate histones. The *T122E* mutation in the H2B C terminal tail may cause the formation of aberrantly condensed chromatin at telomere (perhaps through changes in electrostatic interactions) which may affect gene silencing.

The results presented here show that an appropriately arrayed chromatin mediated by H2B C-terminus is required for optimal SIR binding and the subsequent formation of telomeric chromatin in yeast, which leads to gene silencing. It has been proposed that the BAH domain of Sir3 binds at the gap between nucleosomes in the 11 nm chromatin fiber [14], where the αC helix of H2B facilitates the establishment of internucleosomal contacts [31]. Although the nucleosome has long been assumed to fold into 30 nm chromatin fiber [55], accumulative results from cryo-electron microscopy have not detected 30 nm chromatin fibers in interphase nuclei [8], [13]. This view is supported by a recent paper by Danesh Moazed's laboratory [14]. Moazed and colleagues used a purified system to reconstruct SIR mediated heterochromatin *in vitro*. They observe the formation of extended SIR-nucleosome filaments mediated by the conserved BAH domain in Sir3 [14], indicating that the association of the SIR complex with nucleosome arrays may occur without further chromatin compaction into a 30 nm fiber. Our results suggest that T122 of the H2B C-terminus may be required for its ability to maintain an orderly nucleosome array through inter-nucleosomal contacts (Fig. 7). Therefore, our result supports a model that the SIR complex binds to and spreads along a regularly aligned chromatin fiber that requires the H2B C-terminus.

### H2B C-terminus and chromatin compaction at telomere

We have shown that the residue T122 is critical for silencing and appropriate chromatin structure specifically at the telomere, but it remains unclear as to how the T122E substitution impacts on chromatin structure and accessibility. The crystal structure of yeast nucleosome suggests that H2B T122 faces towards the surface of the nucleosome disk [31] (Fig. 2A), which may contribute to the unique feature of this residue. Through our sucrose gradient sedimentation assay, we show that H2Bub1 and Sir4 are most likely not involved in increasing the mobility of telomeric heterochromatin of *htb1-T122E* (Fig. 3C; Fig. 6C and D). As such, we suggest that perhaps the faster sedimentation of *htb1-T122E* chromatin is induced by altered inter-nucleosomal interactions, resulting in disarrayed and clumped nucleosomes within heterochromatin, causing aberrant compaction (Fig. 7). Confirmation of this hypothesis will necessitate visualization of the chromatin of the mutant strain by electron microscopy, and the modeling of inter-nucleosomal interactions, to determine which residues of H3 interact with H2B C-terminus. However, we cannot completely rule out the possibility that a disruption (whether loss or enhancement) of the levels of H2Bub1 affects telomeric chromatin compaction. The ubiquitylation of H2B is dynamic in nature [34], [37], and these fluctuations may be essential for telomeric chromatin structure, which itself is also flexible and permissive [56], [57], [58].

### A new model for heterochromatin assembly

Our study into the role of the H2B C-terminus in telomeric silencing adds an additional layer of complexity onto the already complex mechanism of heterochromatin silencing. The current model for chromatin assembly at yeast telomere includes initiation, nucleation and spreading [21], [22], [59]. Our results indicate that well arrayed nucleosomes are important for the transition from nucleation to the spreading of silent chromatin at the telomere. We provide evidences that after Rap1 binds to the end of chromosomes (initiation) and the initial round of SIR recruitment is made (nucleation), the SIR complex may require an orderly nucleosome array formed by inter-nucleosomal contacts mediated via the H2B C-terminus to spread into, and subsequently remove acetyl groups from the histone tails of adjacent nucleosomes. Without this prerequisite, the iterative cycle of SIR spreading may not continue, and results in defective assembly of telomeric heterochromatin. We believe that these results are strongly indicative of a real intermediate step in the establishment of telomeric silencing, as despite the *T122E* mutant residue being present in all nucleosomes, other processes on the DNA template (such as transcriptional elongation and DNA repair) appear to be unimpaired (Wang, CY and Kao, CF unpublished data), suggesting that disrupting T122 does not result in change of chromatin structure genome-wide. Additionally, silencing at the mating loci is not impaired, suggesting differences in the mechanisms by which silencing is established at telomeres and mating loci. Differential control of silencing in yeast is supported by a recent study by Sperling and Grunstein (2009), in which they showed that loss of H4 K16 acetylation causes only a slight decrease in silencing at *HMR* locus, despite its overwhelming requirement at the telomere [24], and the finding by Koch and Pillus (2009) that the glucanosyltransferase Gas1 is required for telomeric, but not mating loci silencing [60].

### An intrinsic property of the nucleosome is required for heterochromatin assembly

Various euchromatic marks are associated with the inhibition of SIR binding, to confine the spreading of silenced chromatin [5], [20]. Such euchromatic histone modifications include both acetylation and methylation. Histone acetylation is dynamically regulated [50]; histone methylation is relatively stable, but is still removable via passive dilution through DNA replication or active removal by histone demethylases [50], [61]. On the other hand, the putative inter-nucleosomal interaction mediated by the H2B C-terminus is a stable propensity of chromatin because it is encoded within the protein sequence, independent of H2B ubiquitylation (Fig. 3), and phosphorylation (Table S2). The AVTKY motif of the H2B αC is well-conserved amongst eukaryotes. That implies that, in addition to H2Bub1, this motif plays an unexplored functional role in chromatin structure. Our results suggest that transcriptional gene silencing requires an intrinsic property of the nucleosome, mediated by H2B T122, to form a basal chromatin structure, which is the foundation for the subsequent configuration of heterochromatin at the telomere. This has important implications for our understanding of higher order organization of chromatin *in vivo*.

## Materials and Methods

### Yeast strains and plasmids

Yeast strains used in this study were mainly derived from Y131, UCC6389, and UCC6391 which have been described previously [37]. Yeast cells used were all at log-phase stage. Whole cell extracts were prepared as described [37] and analyzed by western blot using the specific antibodies. Telomere silencing assay was performed with methods as described [39]. All the strains and plasmids are listed in Table S3. Gene disruption and tagging were performed using standard techniques.

### Western blot analysis

Yeast whole cell extracts were prepared as described [37] with minor changes. About 3×10^8^ cells from log-phase yeast cultures were harvested by centrifugation and lyzed in 400 µl of SUME (8 M urea, 1% SDS, 10 mM MOPS, pH 6.8, 10 mM EDTA and 0.01% bromophenol blue) by mechanical shearing using acid-washed glass beads. 10- to 20 µl samples of the lysates were analysed on 15% SDS-polyacrylamide gels, and the proteins were transferred to a PVDF membrane and immunoblotted with the appropriate antibody.

### Telomere length determination

Genomic DNA were extracted, digested with *Xho*I, and separated on 1% agarose gels. After denaturing, DNA fragments were transferred to Hybond N^+^ paper (Amersham Pharmacia Biotech) and then hybridized with a probe containing Y′-telomere sequences.

### RT-PCR analysis

Transcripts for RT-PCR were extracted by the acid-phenol method, purified by use of the Dynabeads® mRNA Purification Kit (Invitrogen) and analyzed by quantitative PCR.

### Pheromone Halo assay

This assay was performed as described [40]. Briefly, small amounts of overnight-cultured cells were mixed well with 0.5% sterile agar at 55°C and then poured onto a pre-warmed plate containing the appropriate solid media. One paper disk containing 5 µl α-factor (5 µg/µl) was placed on the plate and then incubated at 30°C for 16–24 hrs.

### Indirect immunofluorescence microscopy

Indirect immunofluorescence assay was modified from that previously described by Atkin [62]. Briefly, cells were grown to mid-log phase, fixed by 3.7% formaldehyde for 90 mins at 30°C, washed once with 0.1 M potassium phosphate buffer (pH 6.5) and spheroplasted by incubation in spheroplasting buffer [0.2 mg/ml zymolyase 100T (Seikagaku Biobusiness Co., Tokyo, Japan), 10 µl of beta-mercatoethanol per ml of buffer A (0.1 M potassium phosphate buffer, pH 6.5, 1.2 M sorbitol)] at 30°C. The reaction was terminated by washing cells with buffer A. 30 µl of cell suspension was applied to 0.1% poly-_L_-lysine-coated wells, incubated at room temperature for 10 minutes and then washed once with 1× PBS to remove unbound cells. The cells were permeabilized by immersion in methanol at −20°C for 6 mins, followed by immersion in acetone at −20°C for 20 seconds before washing 3 times with 1× PBS. The subsequent blocking, application of primary and secondary antibodies, and treatment of DAPI were carried out as described [62]. Samples were viewed on a confocal laser-scanning microscope LSM 780 (Carl Zeiss, Germany). Images were acquired and processed with ZEN LE software (Carl Zeiss, Germany). Primary antibodies α-myc (for Sir3) (Upstate, MA, USA) were diluted to 1∶200 in PBS- blocking buffer (0.5% BSA, 0.025% NP-40 in PBS buffer). Secondary antibodies Alexa Fluor 488 goat α-mouse IgG (Molecular Probes, CA, USA) were diluted to 1∶500 in PBS-blocking buffer for working concentrations.

### ChIP assay

Chromatin immunoprecipitation in yeast cells was performed as described [63] with modifications. Chromatin solution (500 µl) was incubated with the antibody against Sir2 (Santa Cruz Biotechnology), myc (Millipore), H3 (Abcam), H3 K4me3 (Abcam), H3 K56ac (Millipore), H3 K79me3 (Abcam), H4 K16ac (Millipore), or H4 ac (Millipore) pre-bound to protein-A/G Dynabeads (Invitrogen). After reversal of crosslinking, immunoprecipitated materials were purified by QIAquick PCR purification kit (Qiagen). Samples were assayed by quantitative PCR using the primer pairs listed in Table S4. Each sample was analyzed by three independent experiments. Efficiency of immunoprecipitatons were calculated by dividing the average signal of the eluate by the average signal of the respective input, and normalized with the signal from IntV region. Error bars represent standard deviation.

### Analysis of the genome accessibility to E.coli dam methylase

Yeast strains expressing the *E. coli dam* methylase were grown to stationary phase and genomic DNA was extracted. Samples digested with either DpnI, MboI or Sau3AI (New England Biolabs). Eight to ten units of these enzymes were incubated with 20 µg of the isolated DNA samples for at least 16 h at 37°C, and analyzed by quantitative real-time PCR using a primer pair designed to cross restriction sites at telomere VI-R (shown in Table S4). Results were normalized to DNA content examined by a primer pair for an uncut sequence at *ACT1* gene.

### Sucrose gradient fractionation of chromatin

Sucrose gradient fractionation was performed as described previously [24] with minor modifications. Yeast nuclei were prepared from 1 liter of cells grown to an OD_600_ about 0.8, and 80–100 µg of nuclei was digested by 1,000 U BglII (New England Biolabs). 20%–40% sucrose gradients were prepared by gradient former (Teledyne ISCO), and 200 µL of sample was overlayed on the gradient. After centrifugation at 36,000 rpm in a SW40Ti rotor (Beckman) for 6 h at 4°C, the fractions were collected by Density Gradient Fractionator (Teledyne ISCO), and the relative positions of sub-telomeric (sub-TEL) and euchromatin region (euC) were assayed by quantitative PCR.

### Transcript DNA microarrays

DNA microarray analysis was performed with Phalanx Yeast OneArray® chip (Phalanx Biotech). For each mutant, three independent experiments were performed for the statistical analysis. Yeast genome probe content for the array was selected from Operon Yeast Genome Array-Ready Oligo Set (yeast AROS) v1.1 and Yeast Brown Lab Oligo Extension (YBOX) v1.0. These two sets are 70-mer probes specially designed within 750 bases from the 3′ end of the open reading frame. The fluorescence-labeled probes were hybridized to a chip (Phalanx Biotech) for 16 h at 60°C. After performing the washing steps, the DNA chips were scanned using a ScanArray Lite (PerkinElmer Life Sciences, Billerica, MA, USA). Image analysis was performed with GenePix Pro v 6.0 (Molecular Devices). The raw data were then filtered for signal quality (3 standard deviations above background) and spot quality (minimum diameter). The data were subjected to Lowess normalization with GeneTraffic v 3.2 (Iobion). The data were then exported for input into Cyber-T to assign Bayes *P* values to determine for each ORF whether the mutant was significantly different from the wild type. Changes in relative expression were identified as significant by ranking the Bayesian *P* values and applying a false discovery rate algorithm to account for multiple testing. The false discovery rate threshold was set at 5%. If, for any mutant, an ORF was determined to be significantly different from the wild type, this ORF was included in the cluster analysis. Cluster analysis was performed with Cluster v 2.12 and visualized with Treeview v 1.6. Clusters were analyzed for enrichment of gene classes with FunSpec.

### FLAG-H2B purification by anti-FLAG affinity gel

Log-phase yeast cells grown in YPD medium were treated with H_2_O_2_ (0, 1, 10 mM) for 200 min, caffeine (0, 10 mM) for 30 min, or hydroxyurea 100 mM for 180 min at 30°C, and then harvested by centrifugation. Cells were resuspended in 4 volumes native cracking buffer (1× PBS, 5 mM EGTA, 5 mM EDTA, 0.5% Triton X-100, 0.5% Nonidet-P40, 10% glycerol, 50 mM sodium fluoride, 10 mM β-glycerophosphate, 5 mM sodium pyrophosphate, 5 mM sodium orthovanadate, 1× protease inhibitor cocktail, 2 mM PMSF) and lyzed by mechanical shearing using acid-washed glass beads. After separation from glass beads and centrifugation at 16,000× *g* for 10 min 4°C, the supernatant was incubated with anti-FLAG M2 affinity gel (SIGMA) for 3 h 4°C, and then washed by native cracking buffer and 1× TBS, three times each . Immunoprecipitates were eluted by elution buffer (50 mM Tris-HCl, pH 7.5, 10 mM EDTA, 1% SDS) for 20 min at 42°C.

### Histone purification by acid extraction

Histone extraction by HCl was performed as previously described [64]. Briefly, log-phase yeast cells grown in YPD medium were harvested by centrifugation and washed once with distilled water. After treatment with 8 mg of Zymolyase 100T per gram of cells, spheroplasts were pelleted by centrifugation at 1000× *g* for 5 min, and lyzed in 7 ml/g cells of cold lysis buffer (50 mM MES[KOH], pH 6.0, 75 mM KCl, 0.5 mM CaCl_2_, 0.1% Nonidet-P40, 1 mM PMSF, 1 µM MG132 and 5 µg/ml chymostatin). Pellets containing crude nuclei were resuspended with 7 ml/g cold HS buffer (10 mM MES(KOH), pH 6.0, 430 mM NaCl, 0.5% Nonidet-P40, 1 mM PMSF, 1 µM MG132 and 5 µg/ml chymostatin) for 5 min at 4°C. After centrifugation (15,000× *g*, 5 min), the pellets containing crude chromatin were washed by HS buffer and recovered by centrifugation at 20,000× *g* for 5 min. Histones were extracted from the final pellet with 0.25 M HCl twice, each for 30 min, and then centrifugated 12,000× *g* for 15 min. Histones were recovered by acetone precipitation at −20°C.

### Mass Spectrometry Analyses

Histone H2B was separated from other histones on a 15% SDS-polyacrylamide gel. Trypsin in-gel digestion, liquid chromatography mass spectrometry (LC-MS), and nano LC-MS/MS were adapted from previous work [65]. High-resolution LC-MS/MS experiments were performed on an LTQ-FTICR mass spectrometer (Thermo Electron).

## Supporting Information

Figure S1 The level of H2Bub1 in cells expressing *htb1-T122E* and *htb1-S125E* are comparable with that in *ubp8Δ* strains. Yeast WCEs prepared from the indicated strains were three-fold serial diluted and analyzed by western blot. H2B (Flag-H2B) and its ubiquitylation (Flag-H2Bub1) were detected by anti-Flag antibody.
(TIF)

Figure S2 The levels of histone modifications in H2B C-terminal mutants, including methylation and acetylation at H3 and H4 were not significantly changed. H3 Lys4 mono, di & trimethylation, Lys36 trimethylation, Lys79 trimethylation, and H3 Lys9/14 acetylation and H4 Lys5, 8, 12, 16 acetylation were analyzed using specific antibodies against the modified histones.
(TIF)

Figure S3 The silencing effect at *HML* locus in H2B WT and its mutants. (A) The silencing effect at *HML* locus was measured by the halo assay. Alpha factor inhibits cell growth when silencing is well-maintained. *SIR3* null mutant with silencing defects is shown as a control. (B) The level of gene expression at *HML* locus. Total RNAs were extracted from exponentially growing yeast cells, and mRNAs were purified and analyzed by quantitative PCR using the primer pair *HML* αI. The obtained signals were normalized with the signal from *ACT1*, and then the value of WT was taken as 1 before log transformation. (C) ChIP assay of Sir2 localization at *HML* locus. Primers used are indicated under the gene schematic in part B.
(TIF)

Figure S4 Schematic map of relative locations of the primer pairs used in the sucrose gradient experiments.
(TIF)

Figure S5 The combination of *htb1-S125E* and *RAD6* phosphorylation mutants display mild synthetic defects in telomere silencing. (A) Plasmids carrying *RAD6* or *rad6* mutants were transformed into the strains derived from UCC6389 with *RAD6* deletion expressing *HTB1* or *htb1-S125E*. WCEs prepared from the indicated strains were analyzed by western blot. H2B and its ubiquitylation were detected by anti-Flag antibody; H3 K4 and K79 trimethylation were analyzed by anti-H3 K4me3 or anti-H3 K79me3 antibodies. The G6PDH antibody was used to monitor protein loading. (B) Overnight- cultures of the indicated strains were 5-fold serial diluted and spotted on the indicated plates.
(TIF)

Figure S6 The localization of Sir3 and telomere clustering were strongly affected in *htb1-T122E*, as well as in *SIR4* deletion strains. Strain derived from Y131 or Y131 with *sir4Δ* expressing *HTB1* WT, *htb1-T122E* or *htb-S125E*, were grown to mid-log phase (OD600 = 0.6∼0.8) at 30°C. All strains carry a myc-tagged *SIR3* allele in the genome for monitoring telomere foci. Cells fixed and permeabilized on glass slides were decorated with mouse α-myc monoclonal antibodies (for Sir3) and antibody complexes were later bound with Alexa Fluor 488 goat α-mouse IgG antibodies for visualization. Nuclei were stained with DAPI. Sir3 is in the green channel; DAPI is in the blue channel.
(TIF)

Figure S7 The effects of H2B C-terminal mutants, *htb1-T122E* and *S125E*, on association of Sir3 at telomeric regions. Strains derived from Y131 expressing *HTB1*, *htb1-T122E* and *S125E* were analyzed by chromatin immunoprecipitation using α-myc antibody for Sir3-myc pull-down. DNA samples were then detected by quantitative PCR using the indicated primers.
(TIF)

Figure S8 The levels of SIR proteins were similar between strains expressing H2B WT or mutants. Yeast WCEs were prepared from the Y131-derived strains in which Sir3 and Sir4 were tagged by myc tag. The indicated strains expressing *HTB1* WT or mutants were analyzed by western blot. H2B and its ubiquitylation were detected by anti-Flag antibody; Sir2 was analyzed by α-Sir2 antibody, and Sir3-myc, Sir4-myc were detected by α-myc antibody. The G6PDH antibody was used to monitor the protein loading.
(TIF)

Figure S9 Sir4 deficiency reduces the level of H2Bub1 but not H3 methylations in *htb1-T122E* cells. WCEs of strain UCC6389 or UCC6424 (*sir4Δ*) expressing *HTB1*, *htb1-T122A*, and *htb1-T122E* were analyzed by western blot. H2B and its ubiquitylation were detected by anti-Flag antibody; H3 K4 trimethylation was detected by anti-H3 K4me3 antibodies. The G6PDH antibody was used to monitor protein loading.
(TIF)

Figure S10 The levels of transcripts whose gene products participate in histone ubiquitylation or transcription are not affected in *sir4* deletion mutants. Total RNAs were extracted from Log-phase yeast cells, and mRNAs were purified and analyzed by quantitative PCR. The obtained signals were normalized with the signal from *ACT1*.
(TIF)

Table S1 Systematic mutagenesis of H2B C-terminus.
(DOC)

Table S2 Identification of histone H2B modifications by Mass Spectrometry.
(DOC)

Table S3 Strains and plasmids.
(DOC)

Table S4 Oligonucleotides.
(DOC)
