# Supplementary figures and images for: The C-Terminus of Histone H2B Is Involved in Chromatin Compaction Specifically at Telomeres, Independently of Its Monoubiquitylation at Lysine 123
Source: PLoS One. 2011 Jul 29;6(7):e22209. doi: 10.1371/journal.pone.0022209 (PMC3146481; doi:10.1371/journal.pone.0022209)

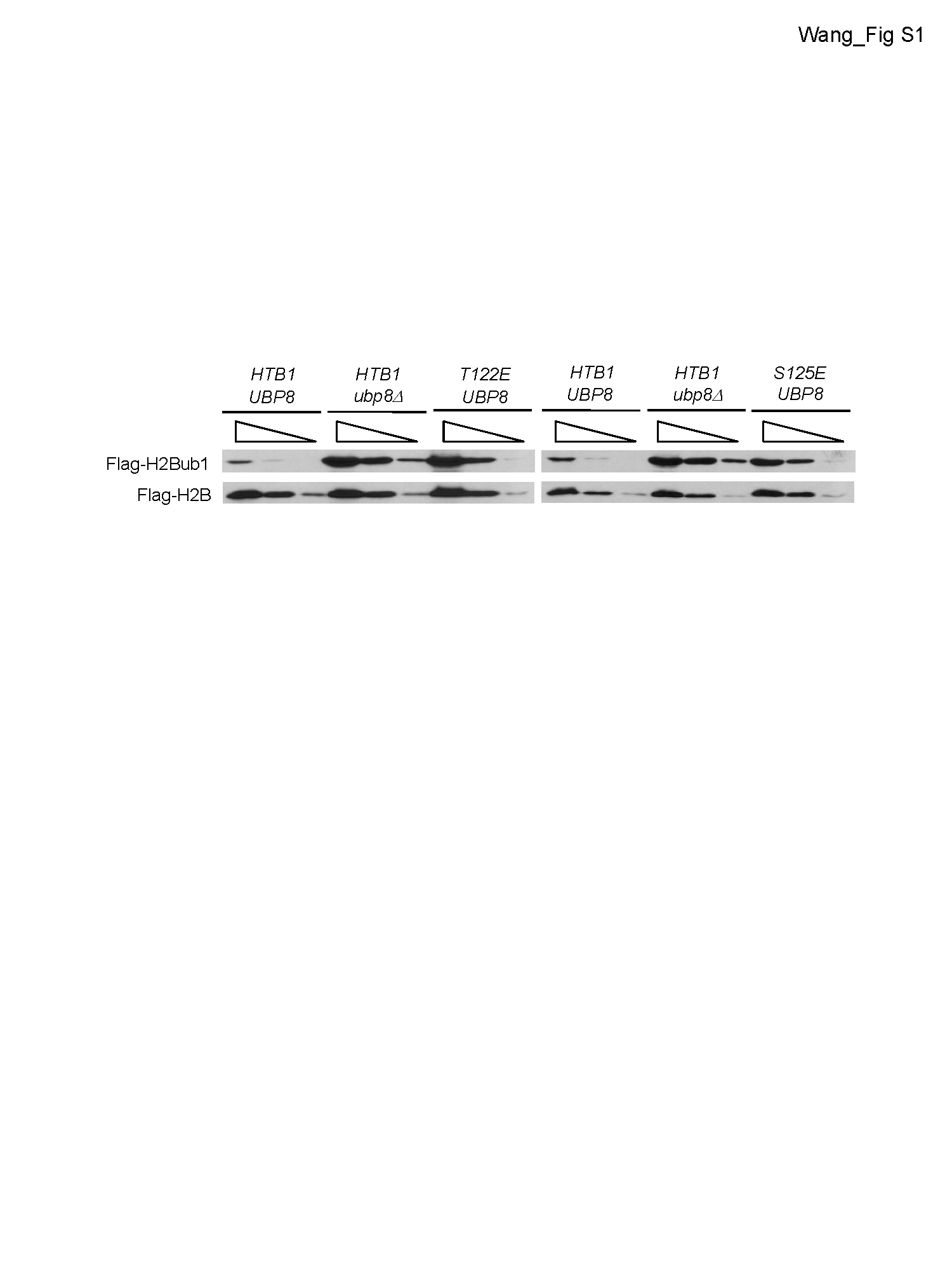

Supplement: Figure S1 — The level of H2Bub1 in cells expressing htb1-T122E and htb1-S125E are comparable with that in ubp8Δ strains. Yeast WCEs prepared from the indicated strains were three-fold serial diluted and analyzed by western blot. H2B (Flag-H2B) and its ubiquitylation (Flag-H2Bub1) were detected by anti-Flag antibody. (TIF) [file pone.0022209.s001.tif]

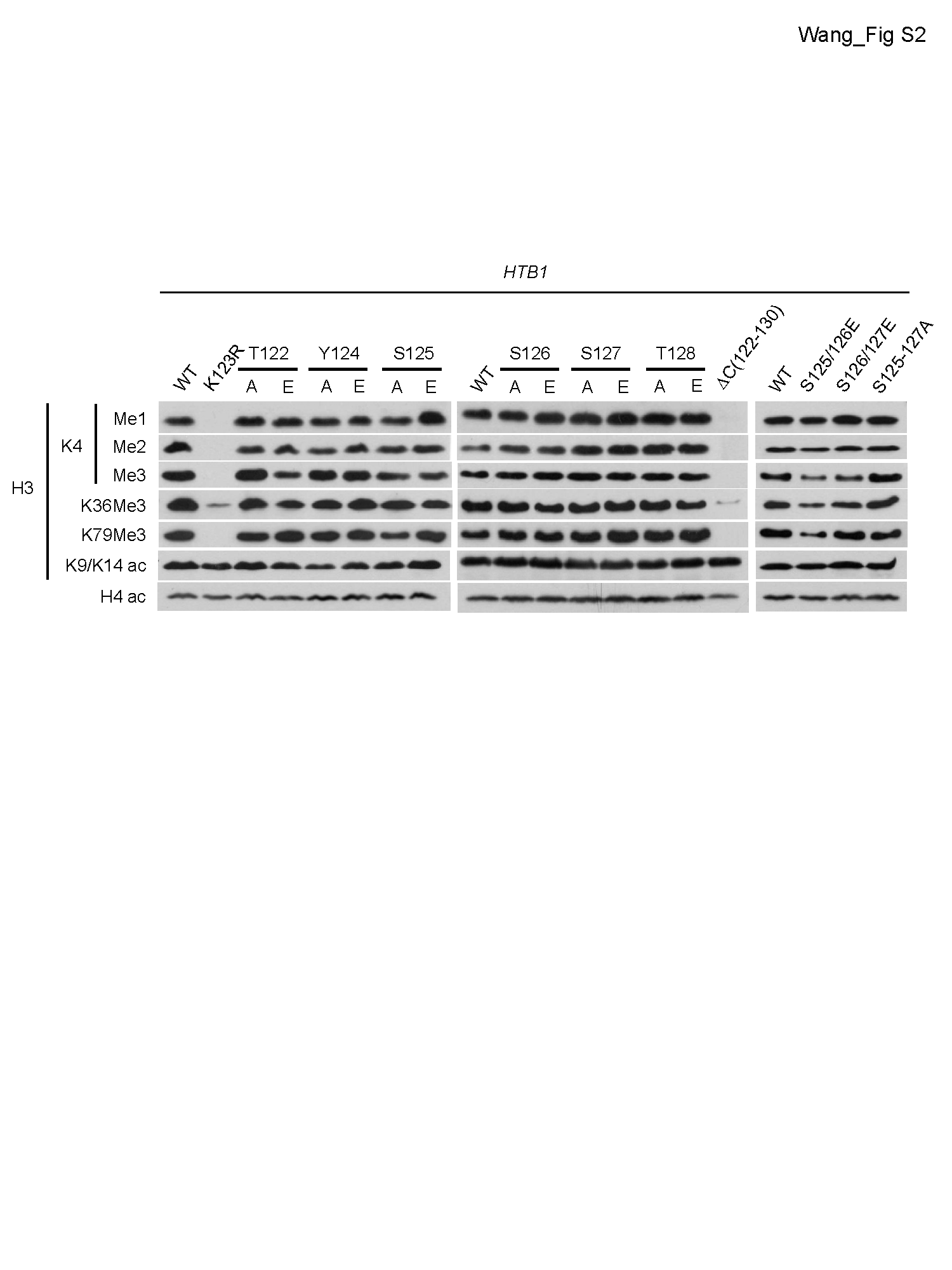

Supplement: Figure S2 — The levels of histone modifications in H2B C-terminal mutants, including methylation and acetylation at H3 and H4 were not significantly changed. H3 Lys4 mono, di & trimethylation, Lys36 trimethylation, Lys79 trimethylation, and H3 Lys9/14 acetylation and H4 Lys5, 8, 12, 16 acetylation were analyzed using specific antibodies against the modified histones. (TIF) [file pone.0022209.s002.tif]

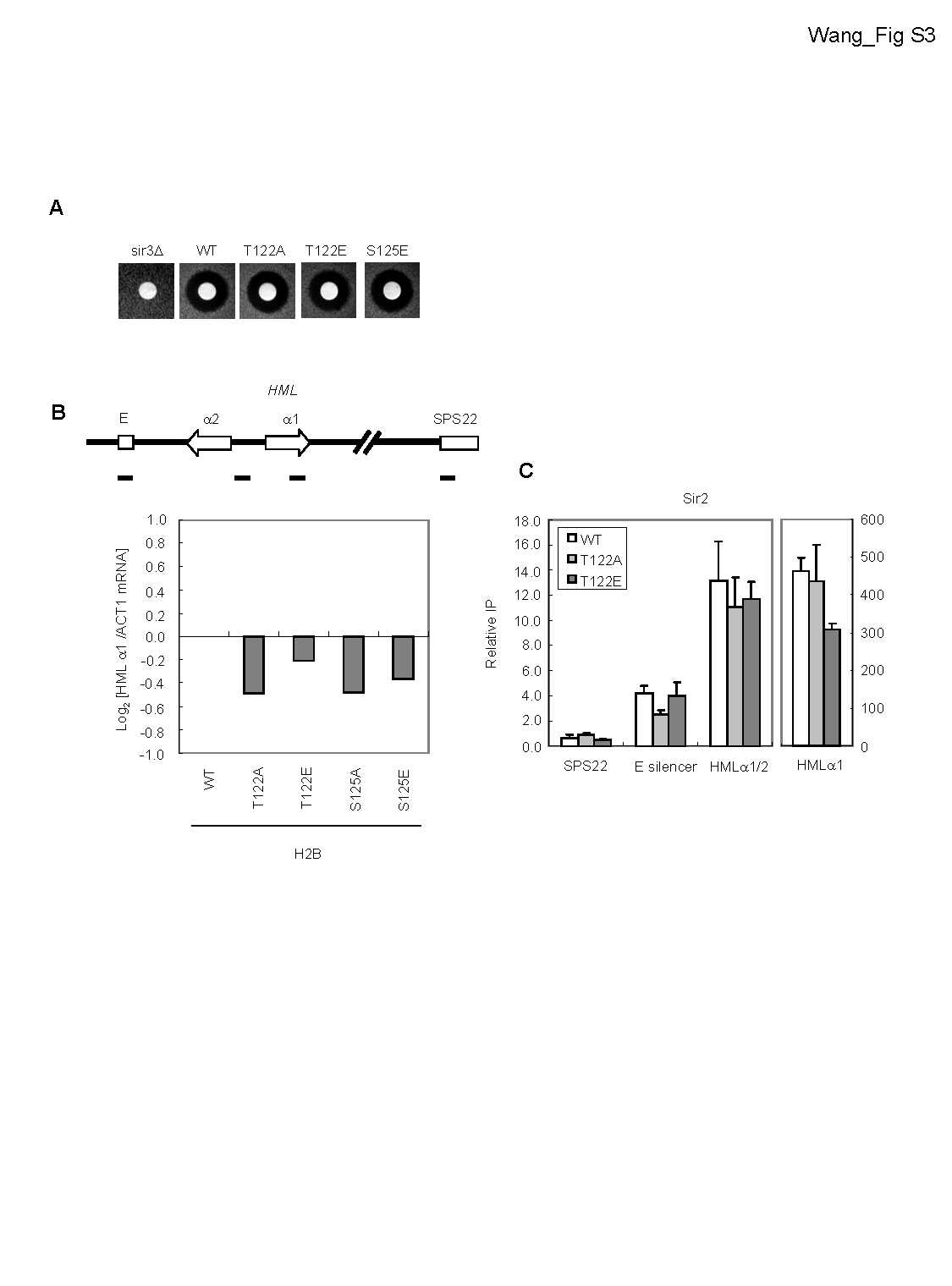

Supplement: Figure S3 — The silencing effect at HML locus in H2B WT and its mutants. (A) The silencing effect at HML locus was measured by the halo assay. Alpha factor inhibits cell growth when silencing is well-maintained. SIR3 null mutant with silencing defects is shown as a control. (B) The level of gene expression at HML locus. Total RNAs were extracted from exponentially growing yeast cells, and mRNAs were purified and analyzed by quantitative PCR using the primer pair HML αI. The obtained signals were normalized with the signal from ACT1, and then the value of WT was taken as 1 before log transformation. (C) ChIP assay of Sir2 localization at HML locus. Primers used are indicated under the gene schematic in part B. (TIF) [file pone.0022209.s003.tif]

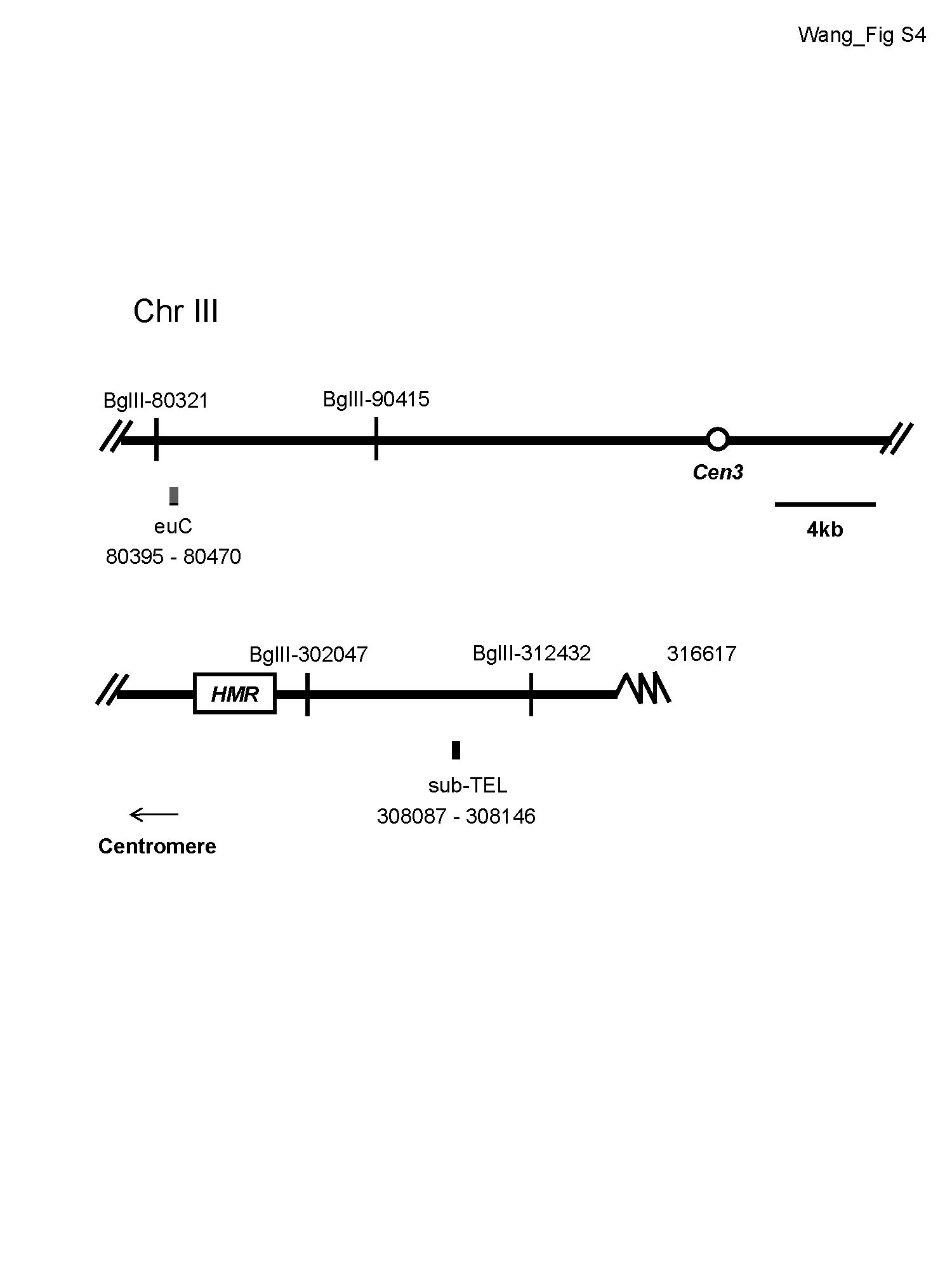

Supplement: Figure S4 — Schematic map of relative locations of the primer pairs used in the sucrose gradient experiments. (TIF) [file pone.0022209.s004.tif]

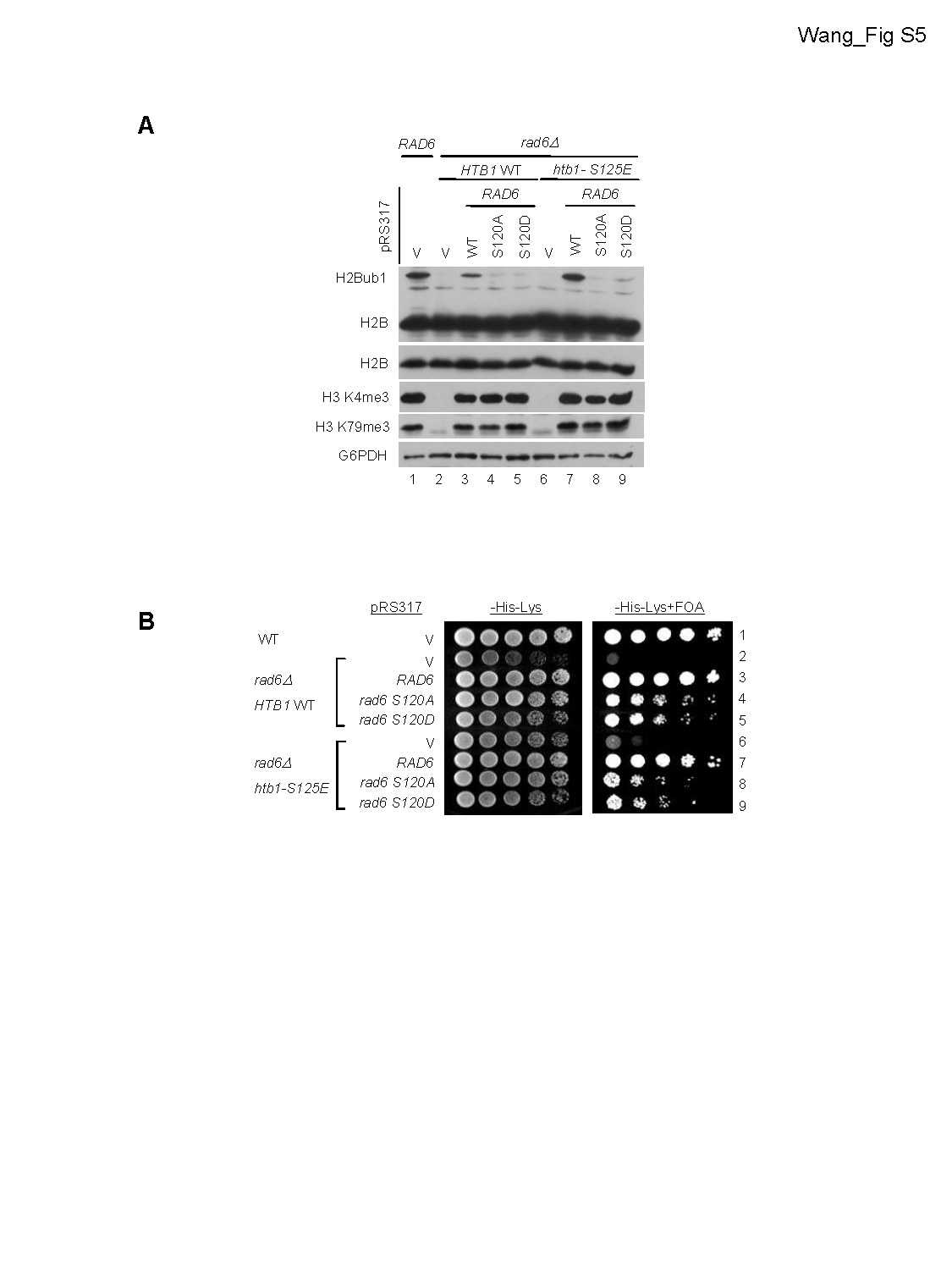

Supplement: Figure S5 — The combination of htb1-S125E and RAD6 phosphorylation mutants display mild synthetic defects in telomere silencing. (A) Plasmids carrying RAD6 or rad6 mutants were transformed into the strains derived from UCC6389 with RAD6 deletion expressing HTB1 or htb1-S125E. WCEs prepared from the indicated strains were analyzed by western blot. H2B and its ubiquitylation were detected by anti-Flag antibody; H3 K4 and K79 trimethylation were analyzed by anti-H3 K4me3 or anti-H3 K79me3 antibodies. The G6PDH antibody was used to monitor protein loading. (B) Overnight- cultures of the indicated strains were 5-fold serial diluted and spotted on the indicated plates. (TIF) [file pone.0022209.s005.tif]

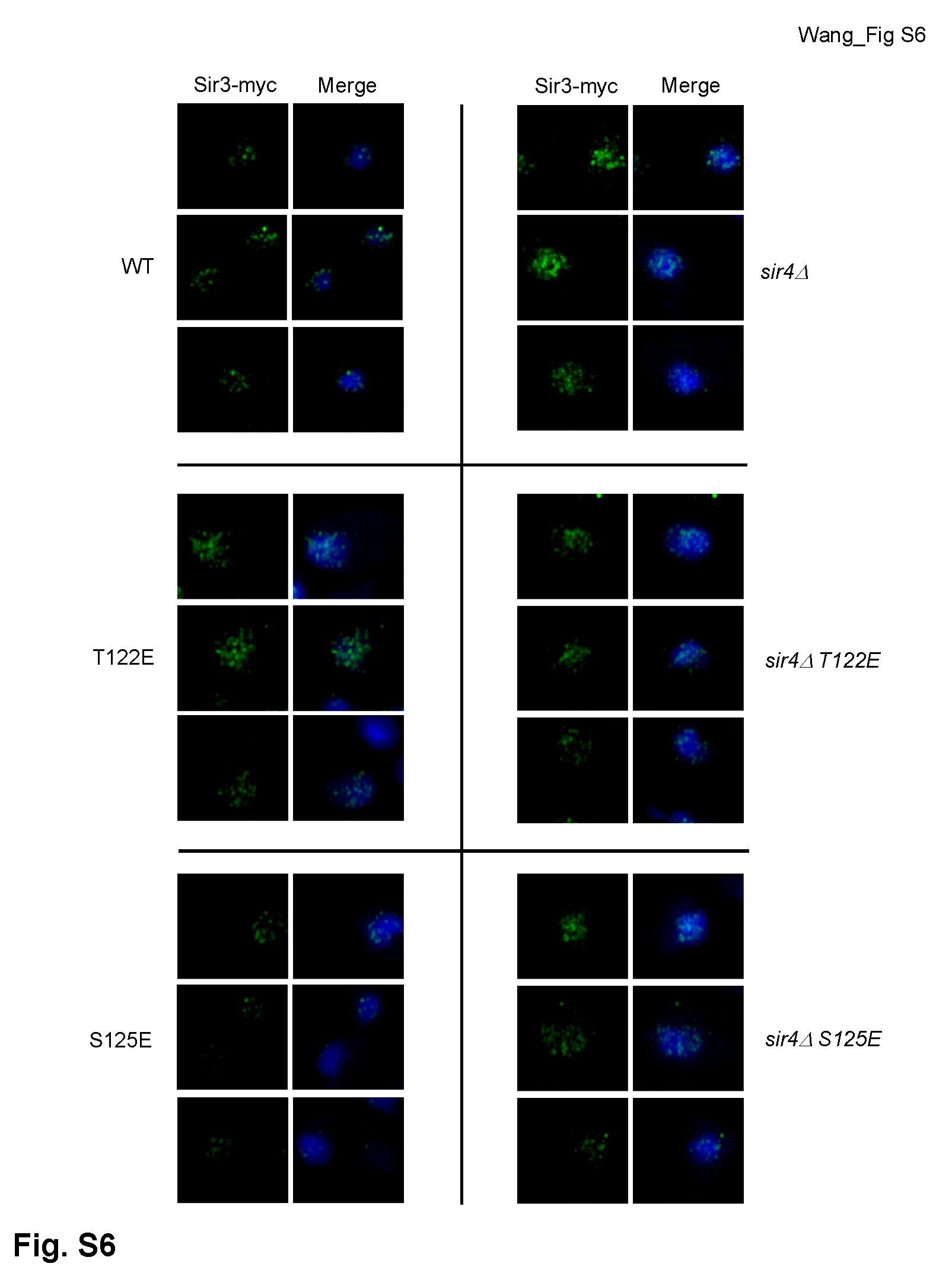

Supplement: Figure S6 — The localization of Sir3 and telomere clustering were strongly affected in htb1-T122E, as well as in SIR4 deletion strains. Strain derived from Y131 or Y131 with sir4Δ expressing HTB1 WT, htb1-T122E or htb-S125E, were grown to mid-log phase (OD600 = 0.6∼0.8) at 30°C. All strains carry a myc-tagged SIR3 allele in the genome for monitoring telomere foci. Cells fixed and permeabilized on glass slides were decorated with mouse α-myc monoclonal antibodies (for Sir3) and antibody complexes were later bound with Alexa Fluor 488 goat α-mouse IgG antibodies for visualization. Nuclei were stained with DAPI. Sir3 is in the green channel; DAPI is in the blue channel. (TIF) [file pone.0022209.s006.tif]

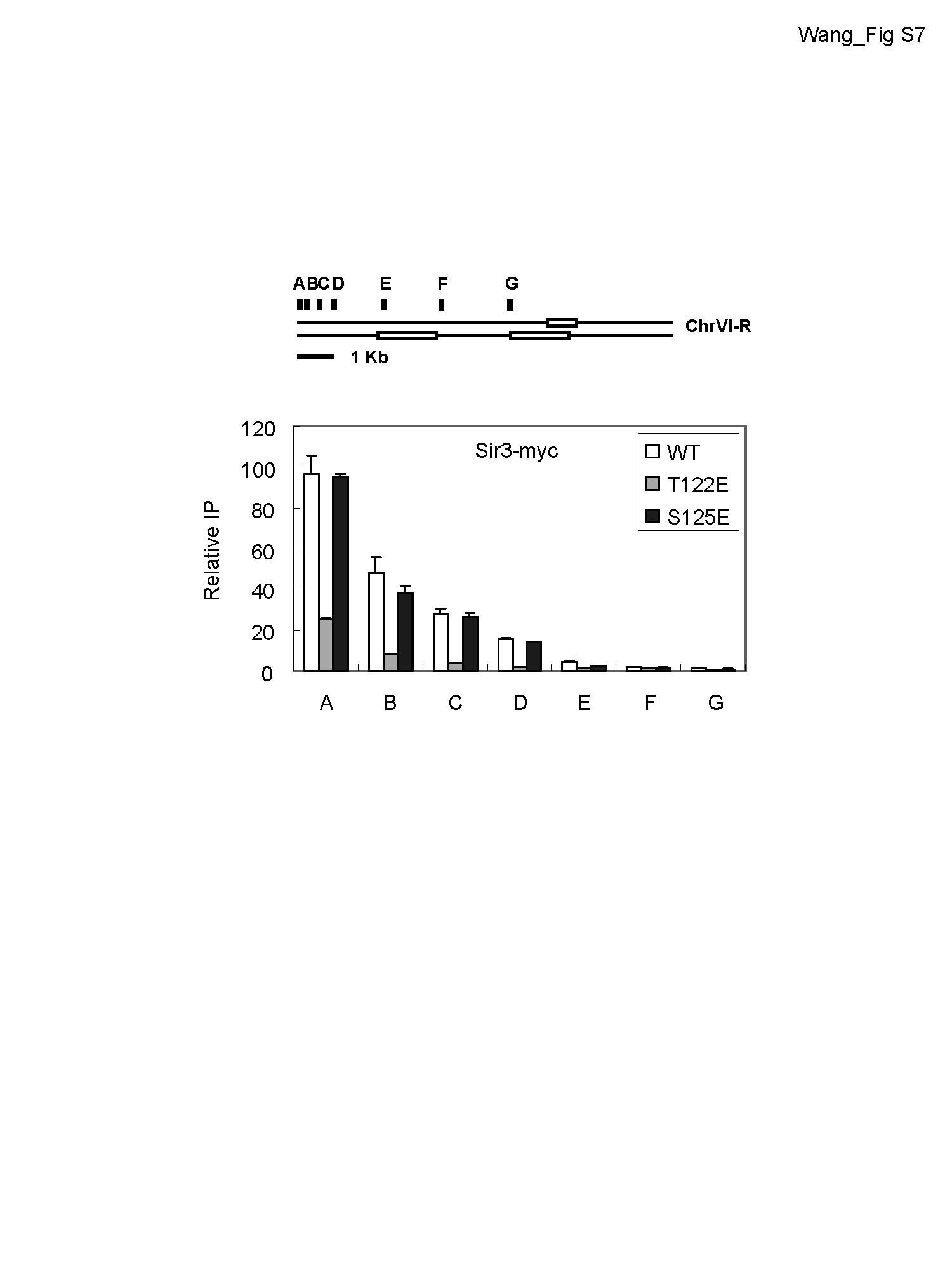

Supplement: Figure S7 — The effects of H2B C-terminal mutants, htb1-T122E and S125E, on association of Sir3 at telomeric regions. Strains derived from Y131 expressing HTB1, htb1-T122E and S125E were analyzed by chromatin immunoprecipitation using α-myc antibody for Sir3-myc pull-down. DNA samples were then detected by quantitative PCR using the indicated primers. (TIF) [file pone.0022209.s007.tif]

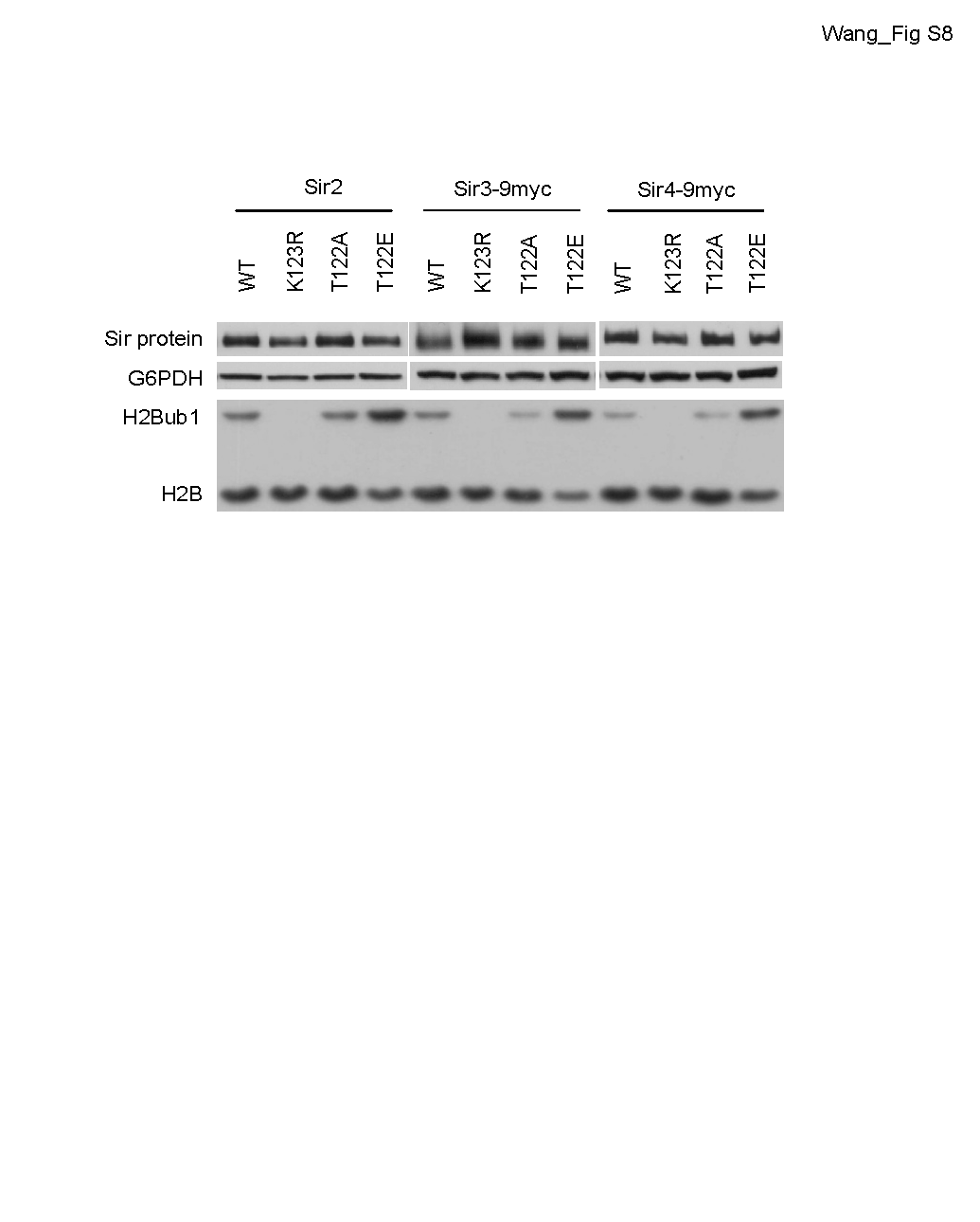

Supplement: Figure S8 — The levels of SIR proteins were similar between strains expressing H2B WT or mutants. Yeast WCEs were prepared from the Y131-derived strains in which Sir3 and Sir4 were tagged by myc tag. The indicated strains expressing HTB1 WT or mutants were analyzed by western blot. H2B and its ubiquitylation were detected by anti-Flag antibody; Sir2 was analyzed by α-Sir2 antibody, and Sir3-myc, Sir4-myc were detected by α-myc antibody. The G6PDH antibody was used to monitor the protein loading. (TIF) [file pone.0022209.s008.tif]

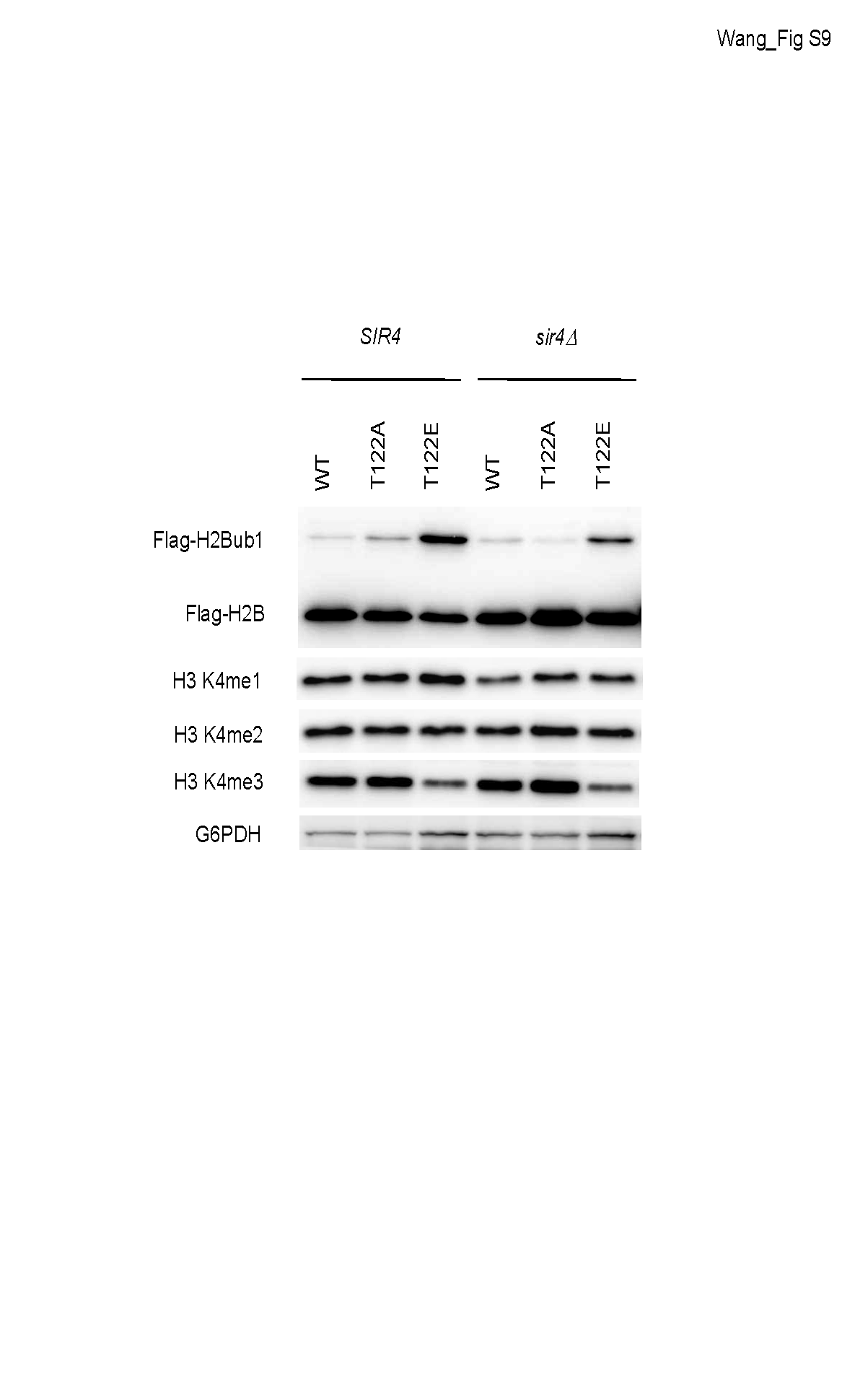

Supplement: Figure S9 — Sir4 deficiency reduces the level of H2Bub1 but not H3 methylations in htb1-T122E cells. WCEs of strain UCC6389 or UCC6424 (sir4Δ) expressing HTB1, htb1-T122A, and htb1-T122E were analyzed by western blot. H2B and its ubiquitylation were detected by anti-Flag antibody; H3 K4 trimethylation was detected by anti-H3 K4me3 antibodies. The G6PDH antibody was used to monitor protein loading. (TIF) [file pone.0022209.s009.tif]

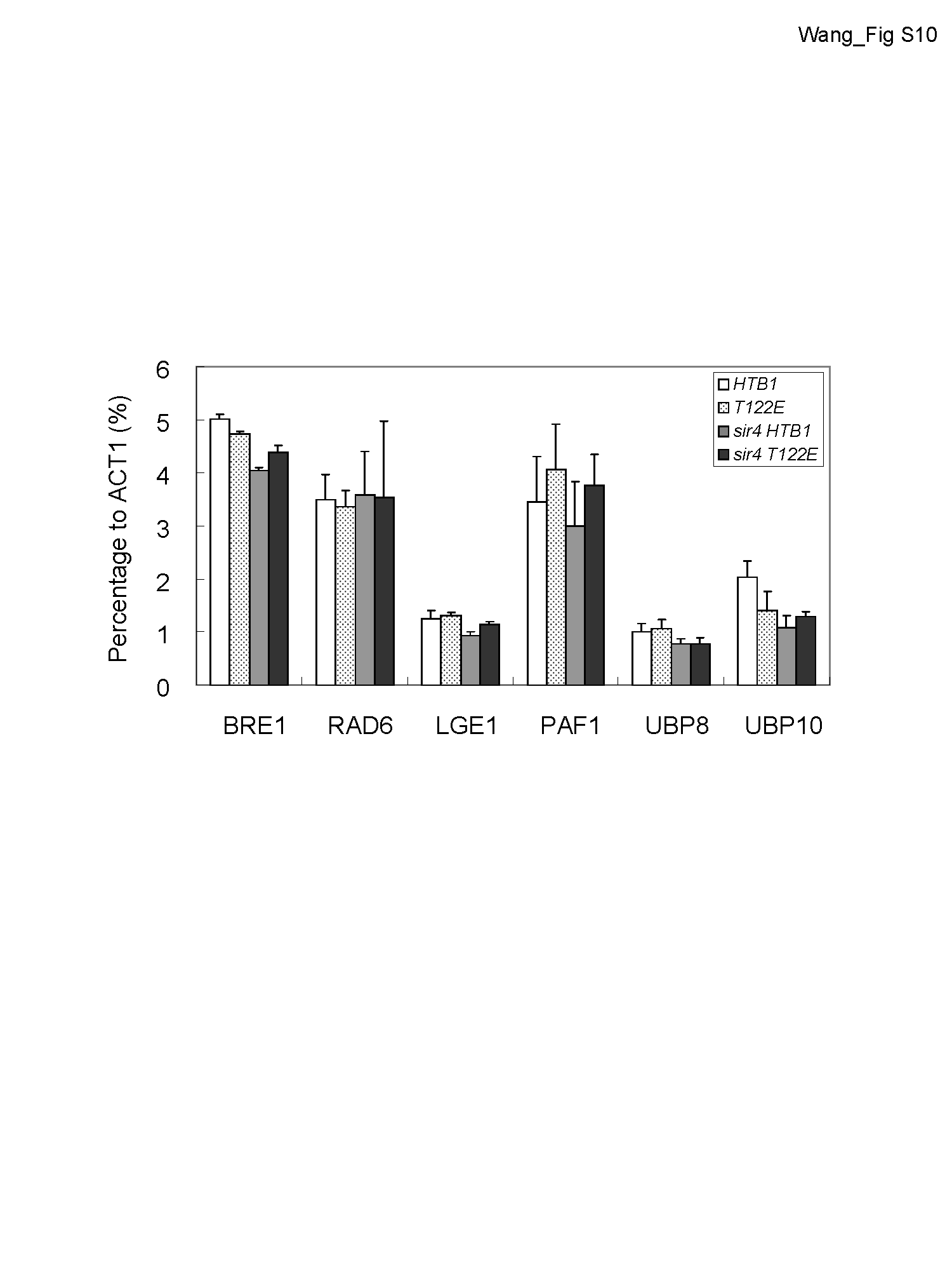

Supplement: Figure S10 — The levels of transcripts whose gene products participate in histone ubiquitylation or transcription are not affected in sir4 deletion mutants. Total RNAs were extracted from Log-phase yeast cells, and mRNAs were purified and analyzed by quantitative PCR. The obtained signals were normalized with the signal from ACT1. (TIF) [file pone.0022209.s010.tif]
