# Supplementary material for: The C-Terminus of Histone H2B Is Involved in Chromatin Compaction Specifically at Telomeres, Independently of Its Monoubiquitylation at Lysine 123
Source: PLoS One. 2011 Jul 29;6(7):e22209. doi: 10.1371/journal.pone.0022209 (PMC3146481; doi:10.1371/journal.pone.0022209)
Supplement: Table S1 — Systematic mutagenesis of H2B C-terminus. (DOC) [file pone.0022209.s011.doc]

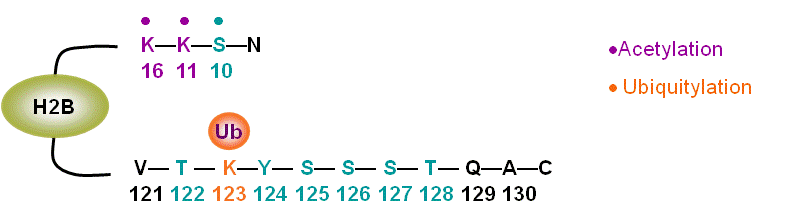


Table S1 Systematic mutagenesis of H2B C-terminus

| **Histone** | **Residue** | **Substitutions** |
| --- | --- | --- |
| H2B | T122 | T122A, T122E |
| H2B | Y124 | Y124A, Y124E |
| H2B | S125 | S125A, S125E |
| H2B | S126 | S126A, S126E |
| H2B | S127 | S127A, S127E |
| H2B | T128 | T128A, T128E |
| H2B | S125, S126 | S125/126E |
| H2B | S126, S127 | S126/127E |
| H2B | S125, S126, S127 | S125-127A |
