# Supplementary material for: The C-Terminus of Histone H2B Is Involved in Chromatin Compaction Specifically at Telomeres, Independently of Its Monoubiquitylation at Lysine 123
Source: PLoS One. 2011 Jul 29;6(7):e22209. doi: 10.1371/journal.pone.0022209 (PMC3146481; doi:10.1371/journal.pone.0022209)
Supplement: Table S2 — Identification of histone H2B modifications by Mass Spectrometry. (DOC) [file pone.0022209.s012.doc]

Table S2 Identification of histone H2B modifications by Mass Spectrometry

| **H2B** | | **Histone extraction** | | |
| --- | --- | --- | --- | --- |
|
|
| **Site** | **Mod** | **-** | **H_2_O_2_** | **HU** |
| **K6** | **Ac** | **X** | **X** | **X** |
| **K7** | **Ac** | **X** |  |  |
| **K11** | **Ac** | **X** | **X** | **X** |
| **K16** | **Ac** | **X** | **X** | **X** |
| **K17** | **Ac** | **X** | **X** | **X** |
| **K21** | **Ac** | **X** | **X** | **X** |
| **K22** | **Ac** | **X** | **X** | **X** |
| **K37** | **Ac** | **X** | **X** |  |
| **K37** | **Me** | **X** | **X** | **X** |
| **T39** | **P** | **X** | **X** | **X** |
| **K49** | **Ac** | **X** | **X** |  |
| **K88** | **Ac** |  | **X** |  |
| **K111** | **Me** |  | **X** |  |
| **K123** | **Ub** |  | **X** | **X** |
| **T128** | **P** | **X** | **X** |  |

**X** indicates that the modification has been detected at least two times.
