# Supplementary material for: The C-Terminus of Histone H2B Is Involved in Chromatin Compaction Specifically at Telomeres, Independently of Its Monoubiquitylation at Lysine 123
Source: PLoS One. 2011 Jul 29;6(7):e22209. doi: 10.1371/journal.pone.0022209 (PMC3146481; doi:10.1371/journal.pone.0022209)
Supplement: Table S3 — Strains and plasmids. (DOC) [file pone.0022209.s013.doc]

Table S3 Strains and plasmids

| strains and  plasmids | Genotype or description | source or reference |
| --- | --- | --- |
|
| **Yeast strains** |  |  |
| Y131 | *MAT***a** *ura3-1 leu2-3,-112 ade2-1 trp1-1 his3-11,15 can1-100 hta1-htb1::LEU2, hta2-htb2* pRS426-TRT1 | Sun and Allis, 2002 |
|
| YZS276 | as Y131, pRS426-TRT1 replaced by pZS145 | this study |
| YZS277 | as Y131, pRS426-TRT1 replaced pZS146 | this study |
| YCW100 | as Y131, pRS426-TRT1 replaced pCK-T122A | this study |
| YCW101 | as Y131, pRS426-TRT1 replaced pCK-T122E | this study |
| YCW102 | as Y131, pRS426-TRT1 replaced pCK-Y124A | this study |
| YCW103 | as Y131, pRS426-TRT1 replaced pCK-Y124E | this study |
| YCW104 | as Y131, pRS426-TRT1 replaced pCK-S125A | this study |
| YCW105 | as Y131, pRS426-TRT1 replaced pCK-S125E | this study |
| YCW106 | as Y131, pRS426-TRT1 replaced pCK-S126A | this study |
| YCW107 | as Y131, pRS426-TRT1 replaced pCK-S126E | this study |
| YCW108 | as Y131, pRS426-TRT1 replaced pCK-S127A | this study |
| YCW109 | as Y131, pRS426-TRT1 replaced pCK-S127E | this study |
| YCW110 | as Y131, pRS426-TRT1 replaced pCK-T128A | this study |
| YCW111 | as Y131, pRS426-TRT1 replaced pCK-T128E | this study |
| YCW112 | as Y131, pRS426-TRT1 replaced pCK-S125/126E | this study |
| YCW113 | as Y131, pRS426-TRT1 replaced pCK-S126/127E | this study |
| YCW114 | as Y131, pRS426-TRT1 replaced pCK-S125/126/127A | this study |
| YCW115 | as Y131, pRS426-TRT1 replaced pCK-ΔC(122-130) | this study |
| UCC6389 pRG422 | *MAT***a** *lys2Δ0 trp1Δ63 his3Δ200 ade2Δ*::*hisG ura3Δ0 leu2Δ0 met15Δ0 ADE2-TEL-VR URA3-TEL-VIIL htaI-htb1*::*MET15 hta2-htb2*::*LEU2* pRG422 | Gardner et al., 2005 |
|
| YCW116AU | as UCC6389, pRG422 replaced by pRS317-TRT1 | this study |
| YCW117AU | as UCC6389, pRG422 replaced by pZS145 | this study |
| YCW118AU | as UCC6389, pRG422 replaced by pZS146 | this study |
| YCW119AU | as UCC6389, pRG422 replaced by pCK-T122A | this study |
| YCW120AU | as UCC6389, pRG422 replaced by pCK-T122E | this study |
| YCW121AU | as UCC6389, pRG422 replaced by pCK-Y124A |  |
| YCW122AU | as UCC6389, pRG422 replaced by pCK-Y124E |  |
| YCW123AU | as UCC6389, pRG422 replaced by pCK-S125A | this study |
| YCW124AU | as UCC6389, pRG422 replaced by pCK-S125E | this study |
| YCW125AU | as UCC6389, pRG422 replaced by pCK-S126A | this study |
| YCW126AU | as UCC6389, pRG422 replaced by pCK-S126E | this study |
| YCW127AU | as UCC6389, pRG422 replaced by pCK-S127A | this study |
| YCW128AU | as UCC6389, pRG422 replaced by pCK-S127E | this study |
| YCW129AU | as UCC6389, pRG422 replaced by pCK-T128A | this study |
| YCW130AU | as UCC6389, pRG422 replaced by pCK-T128E | this study |
| YCW131AU | as UCC6389, pRG422 replaced by pCK-S125/126E | this study |
| YCW132AU | as UCC6389, pRG422 replaced by pCK-S126/127E | this study |
| YCW133AU | as UCC6389, pRG422 replaced by pCK-S125/126/127A | this study |
| UCC6391 pRG422 | UCC6389 *sir4Δ*::*KanMX* pRG422 | Gardner et al., 2005 |
| YCW134AU | as UCC6391, pRG422 replaced by pZS145 | this study |
| YCW135AU | as UCC6391, pRG422 replaced by pCK-T122E | this study |
| YCW136AU | UCC6389 *rad6Δ*::*KanMX,* pRG422 replaced by pZS145 | this study |
| YCW137AU | UCC6389 *rad6Δ*::*KanMX,* pRG422 replaced by pCK-T122E | this study |
| YCW138AU | UCC6389 *rad6Δ*::*KanMX,* pRG422 replaced by pCK-S125E | this study |
| YCW139 | Y131 *pep4Δ::HPH RAP1-13myc::KAN,* pRS426-TRT1 *replaced by* pZS145 | this study |
| YCW140 | Y131 *pep4Δ::HPH RAP1-13myc::KAN,* pRS426-TRT1 *replaced by* pCK-T122A | this study |
| YCW141 | Y131 *pep4Δ::HPH RAP1-13myc::KAN,* pRS426-TRT1 *replaced by* pCK-T122E | this study |
| YCW006 | Y131 *pep4Δ::HPH SIR3-myc::natMX,* pRS426-TRT1 *replaced by* pZS145 | this study |
| YCW007 | Y131 *pep4Δ::HPH SIR3-myc::natMX,* pRS426-TRT1 *replaced by* pZS146 | this study |
| YCW008 | Y131 *pep4Δ::HPH SIR3-myc::natMX,* pRS426-TRT1 *replaced by* pCK-T122A | this study |
| YCW009 | Y131 *pep4Δ::HPH SIR3-myc::natMX,* pRS426-TRT1 *replaced by* pCK-T122E | this study |
| YCW010 | Y131 *pep4Δ::HPH SIR3-myc::natMX,* pRS426-TRT1 *replaced by* pCK-S125E | this study |
| YCW011 | Y131 *pep4Δ::HPH SIR4-myc::natMX,* pRS426-TRT1 *replaced by* pZS145 | this study |
| YCW012 | Y131 *pep4Δ::HPH SIR4-myc::natMX,* pRS426-TRT1 *replaced by* pZS146 | this study |
| YCW013 | Y131 *pep4Δ::HPH SIR4-myc::natMX,* pRS426-TRT1 *replaced by* pCK-T122A | this study |
| YCW014 | Y131 *pep4Δ::HPH SIR4-myc::natMX,* pRS426-TRT1 *replaced by* pCK-T122E | this study |
| YCW096 | Y131 *pep4Δ::HPH SIR3-9myc::natMX sir4Δ::KANMX*, pRS426-TRT1 replaced by pZS145 | this study |
| YCW097 | Y131 *pep4Δ::HPH SIR3-9myc::natMX sir4Δ::KANMX*, pRS426-TRT1 replaced by pCK-T122E | this study |
| YCW098 | Y131 *pep4Δ::HPH SIR3-9myc::natMX sir4Δ::KANMX*, pRS426-TRT1 replaced by pCK-S125E | this study |
| YCW099 | Y131 *ubp8Δ::KAN*, pRS426-TRT1 replaced by pZS145 | this study |
| YCW142AU | UCC6389 *lys2Δ0::dam^+^::LYS2*, pRG422 replaced by pZS145 | this study |
| YCW143AU | UCC6389 *lys2Δ0::dam^+^::LYS2*, pRG422 replaced by pCK-T122A | this study |
| YCW144AU | UCC6389 *lys2Δ0::dam^+^::LYS2*, pRG422replaced by pCK-T122E | this study |
| YCW145AU | UCC6389 *lys2Δ0::dam^+^::LYS2*, pRG422 replaced by pCK-S125E | this study |
| YCW146AU | UCC6391 *lys2Δ0::dam^+^::LYS2*, pRG422 replaced by pCK-S126E | this study |
|  |  |  |
| **Plasmids** |  |  |
| pRS426-TRT1 | *URA3 2μ HTA1-HTB1* | Sun and Allis, 2002 |
| pZS145 | *HIS3 CEN HTA1-Flag-HTB1* | Sun and Allis, 2002 |
| pZS146 | *HIS3 CEN HTA1-Flag-htb1K123R* | Sun and Allis, 2003 |
| pCK-T122A | *HIS3 CEN HTA1-Flag-htb1T122A* | this study |
| pCK-T122E | *HIS3 CEN HTA1-Flag-htb1T122E* | this study |
| pCK-Y124A | *HIS3 CEN HTA1-Flag-htb1Y124A* | this study |
| pCK-Y124E | *HIS3 CEN HTA1-Flag-htb1Y124E* | this study |
| pCK-S125A | *HIS3 CEN HTA1-Flag-htb1S125A* | this study |
| pCK-S125E | *HIS3 CEN HTA1-Flag-htb1S125E* | this study |
| pCK-S126A | *HIS3 CEN HTA1-Flag-htb1S126A* | this study |
| pCK-S126E | *HIS3 CEN HTA1-Flag-htb1S126E* | this study |
| pCK-S127A | *HIS3 CEN HTA1-Flag-htb1S127A* | this study |
| pCK-S127E | *HIS3 CEN HTA1-Flag-htb1S127E* | this study |
| pCK-T128A | *HIS3 CEN HTA1-Flag-htb1T128A* | this study |
| pCK-T128E | *HIS3 CEN HTA1-Flag-htb1T128E* | this study |
| pRS317 | *Lys2 CEN* | Eriksson et al., 2004 |
| pRS317-TRT1 | *Lys2 CEN HTA1-Flag-HTB1* | this study |
| pRS317-RAD6 | *Lys2 CEN RAD6* | this study |
| pRS317-rad6 S120A | *Lys2 CEN rad6 S120A* | this study |
| pRS317-rad6 S120D | *Lys2 CEN rad6 S120D* | this study |

Reference:

Eriksson, P., Thomas, L. R., Thorburn, A., and Stillman, D. J., pRS yeast vectors with a LYS2 marker. *Biotechniques* **36** (2), 212 (2004).

Kao, C. F. et al., Rad6 plays a role in transcriptional activation through ubiquitylation of histone H2B. *Genes Dev* **18** (2), 184 (2004).
