# Supplementary material for: The C-Terminus of Histone H2B Is Involved in Chromatin Compaction Specifically at Telomeres, Independently of Its Monoubiquitylation at Lysine 123
Source: PLoS One. 2011 Jul 29;6(7):e22209. doi: 10.1371/journal.pone.0022209 (PMC3146481; doi:10.1371/journal.pone.0022209)
Supplement: Table S4 — Oligonucleotides. (DOC) [file pone.0022209.s014.doc]

Table S4 Oligonucleotides

| **Oligonucleotides** | **Description** | **Source or reference** |
| --- | --- | --- |
|  |  |  |
| TEL6R-012F | CGTGTGTAGTGATCCGAACTCAGT | this study |
| TEL6R-012R | GACCAGTCCTCATTTCCATCAATAG | this study |
| TEL6R-036F | GTTTAACGGTGATTATTAGGTGG | this study |
| TEL6R-036R | GCGTTATGACAATTTTATGTAGATATCC | this study |
| TEL6R-066F | CGTAACAAAGCCATAATGCC | this study |
| TEL6R-066R | CAGAAAGTAGTCCAGCCG | this study |
| TEL6R-099F | GTCTATAGTAAGTGCTCGGC | this study |
| TEL6R-099R | AACATAACTTTGATCCTTACTCG | this study |
| TEL6R-2480F | GACAGGTCCATTTATCATCCAG | this study |
| TEL6R-2480R | CAATGAATCTTCGGTGCTT | this study |
| TEL6R-4000F | TCTCGGTGAACGGATGC | this study |
| TEL6R-4000R | TTCCTTATAACCTTTTTTACACGACA | this study |
| TEL6R-5990F | GACTGCTCTAGTGTTGGG | this study |
| TEL6R-5990R | TGAATATTTTGGTTTTTGCTGGAA | this study |
| Int-V-F | TAAGAGGTGATGGTGATAGGCGT | Kao et al., 2004 |
| Int-V-R | CCC TCG GGT CAA ACA CTA CAC | Kao et al., 2004 |
| HMLalpha1-F | GCTGAAGAATGGCACGC | this study |
| HMLalpha1-R | TCTACAAAACCAAAACCAGGG | this study |
| sub-TEL-F | GTGTCCCATGGTACTCCACTAGA | this study |
| sub-TEL-R | CGTCCTTAGATAGAGCACTGGAG | this study |
| euC-F | ACATCCAAGAGGCATTCCAG | this study |
| euC-R | CGGTAGGATTCGAGGACAGT | this study |
| BRE1-F | AATTAAGCGATCCTAGTGAACC | this study |
| BRE1-R | TATTTGCCAACTTGCGGG | this study |
| RAD6-F | AGAAGAAGGTTGATGAGAGATTT | this study |
| RAD6-R | CGGTGGCTTATTGGGATATT | this study |
| PAF1-F | ACGCACCGAATATGTCT | this study |
| PAF1-R | GTTGCCATTTGTCCGTC | this study |
| LGE1-F | GCGGATACACGGGAAAT | this study |
| LGE1-R | GCGGCTGCTCATAGTCA | this study |
| UBP8-F | GGAACCATTCGCATTTTCT | this study |
| UBP8-R | TCGTCCCAATACTTCGCT | this study |
| UBP10-F | ACAACGCAGATAAGGGC | this study |
| UBP10-R | TCGTCGTCTTCTATTTCGTCA | this study |
| TEL6R-dam-F | GCTGAGATAAGTAATATCGTTGATGAA | this study |
| TEL6R-dam-R | TCAAACAAGTAGGAATGCGAAAG | this study |
| ACT1-dam-F | AGGTTGCTGCTTTGGTTA | this study |
| ACT1-dam-R | CGTAGGAGTCTTTTTGACCC | this study |
| SPS22-F | TTGGGTGCCGCTATTGAT | this study |
| SPS22-R | ACGGTTCATTTGCTCTTCT | this study |
| HML-E-F | GAATCAAATAGGTGTATCGCAATGG | Xu et al., 2007 |
| HML-E-R | GTTAGATTTGGCCCCCGAAATCG | Xu et al., 2007 |
| HML-I-F | AACTTACTTCAACATGAAAGCCCG | Xu et al., 2007 |
| HML-I-R | AAGAACGTACATAGTGTGCCCAGC | Xu et al., 2007 |
